# An Advanced Preclinical Mouse Model for Acute Myeloid Leukemia Using Patients' Cells of Various Genetic Subgroups and *In Vivo* Bioluminescence Imaging

**DOI:** 10.1371/journal.pone.0120925

**Published:** 2015-03-20

**Authors:** Binje Vick, Maja Rothenberg, Nadine Sandhöfer, Michela Carlet, Cornelia Finkenzeller, Christina Krupka, Michaela Grunert, Andreas Trumpp, Selim Corbacioglu, Martin Ebinger, Maya C. André, Wolfgang Hiddemann, Stephanie Schneider, Marion Subklewe, Klaus H. Metzeler, Karsten Spiekermann, Irmela Jeremias

**Affiliations:** 1 Group Apoptosis, Department of Gene Vectors, Helmholtz Zentrum München, German Research Center for Environmental Health, Munich, Germany; 2 German Cancer Consortium (DKTK), Heidelberg, Germany; 3 German Cancer Research Center (DKFZ), Heidelberg, Germany; 4 Department of Internal Medicine III, University Hospital Grosshadern, Ludwig-Maximilians-Universität (LMU), Munich, Germany; 5 Clinical Cooperation Group Leukemia, Helmholtz Zentrum München, German Research Center for Environmental Health, Munich, Germany; 6 Clinical Cooperation Group Immunotherapy, Helmholtz Zentrum München, German Research Center for Environmental Health, Munich, Germany; 7 Division of Stem Cells and Cancer, German Cancer Research Center (DKFZ), Heidelberg, Germany; 8 Heidelberg Institute for Stem Cell Technology and Experimental Medicine (HI-STEM GmbH), Heidelberg, Germany; 9 Department of Pediatrics, University of Regensburg, Regensburg, Germany; 10 Department of Pediatric Hematology/Oncology, University Children’s Hospital, Eberhard Karls Universität, Tuebingen, Germany; 11 Department of Pediatric Intensive Care Medicine, University Children's Hospital (UKBB), Basel, Switzerland; 12 Department of Oncology, Dr von Haunersches Kinderspital, Ludwig Maximilians-Universität (LMU), Munich, Germany; Università degli Studi di Firenze, ITALY

## Abstract

Acute myeloid leukemia (AML) is a clinically and molecularly heterogeneous disease with poor outcome. Adequate model systems are required for preclinical studies to improve understanding of AML biology and to develop novel, rational treatment approaches. Xenografts in immunodeficient mice allow performing functional studies on patient-derived AML cells. We have established an improved model system that integrates serial retransplantation of patient-derived xenograft (PDX) cells in mice, genetic manipulation by lentiviral transduction, and essential quality controls by immunophenotyping and targeted resequencing of driver genes. 17/29 samples showed primary engraftment, 10/17 samples could be retransplanted and some of them allowed virtually indefinite serial transplantation. 5/6 samples were successfully transduced using lentiviruses. Neither serial transplantation nor genetic engineering markedly altered sample characteristics analyzed. Transgene expression was stable in PDX AML cells. Example given, recombinant luciferase enabled bioluminescence *in vivo* imaging and highly sensitive and reliable disease monitoring; imaging visualized minimal disease at 1 PDX cell in 10000 mouse bone marrow cells and facilitated quantifying leukemia initiating cells. We conclude that serial expansion, genetic engineering and imaging represent valuable tools to improve the individualized xenograft mouse model of AML. Prospectively, these advancements enable repetitive, clinically relevant studies on AML biology and preclinical treatment trials on genetically defined and heterogeneous subgroups.

## Introduction

Acute myeloid leukemia (AML) remains a hematologic malignancy with poor outcome. There is a strong demand for preclinical models to develop novel, targeted therapies based on a better understanding of the complex biology of AML [1]. Although a multitude of tumor cell lines exist [2], important functional characteristics may be altered in cell lines during the process of immortalization [3]. Work with primary tumor cells represents an attractive alternative more closely related to the patient, but primary AML cells rarely show sustained growth *in vitro*, despite improvements using cytokine-based co-culture systems [4].

To allow *in vivo* studies on AML, engraftment of patient-derived cells in immuno-compromised mice has been established in the early 1990s using severe combined immunodeficiency (scid) mice [5–7]. Engraftment capacity had improved by using more severely immuno-compromised mice such as the Non-Obese Diabetic (NOD)/scid mice [8–12] and lately NOD/scid IL2 receptor gamma chain knockout (NSG) mice, which virtually lack B, T, and functional NK cells [13–19].

Using the individualized xenograft mouse model of AML has resulted in seminal insights, e.g., in stem cell biology [20,21]; nevertheless, the model has not yet been used extensively for other purposes. First, most studies were performed on mice injected with primary patient cells, while retransplantation of engrafted AML cells was restricted to studies analyzing stem cell features and self-renewing capacity [9,10,17,19]. Nevertheless, serial retransplantation is highly attractive as it provides a continuous supply of patient-derived xenograft (PDX) AML cells for repetitive functional and therapeutic studies both *in vitro* and *in vivo*, but this approach has not yet been systematically explored. Second, genetic engineering was never reported in established PDX AML cells. Molecular studies were mainly restricted to AML cell lines, which were genetically altered both for *in vitro* and *in vivo* studies, and both for knockdown strategies and transgene overexpression, including *in vivo* imaging [22–24]. Nevertheless, PDX AML cells represent a highly interesting tool for molecular studies, e.g., on signaling proteins, due to their close relationship to the patient sample, in contrast to established AML cell lines. Thirdly, monitoring the growth characteristics of PDX cells *in vivo* is an important readout for preclinical studies, yet this remains challenging as PDX cells are detected in mouse peripheral blood (PB) only at late disease stages using flow cytometry or polymerase chain reaction [16,17,19], and repetitive bone marrow (BM) aspirations are performed infrequently for animal welfare [25,26]. Analysis of murine inner organs like spleen, liver, and kidney can only be performed post mortem [16,17,19], which constitutes a major disadvantage in preclinical treatment trials [27].

Serial passaging and genetic engineering have already been established in studies using primary tumor cells from patients with acute lymphoblastic leukemia (ALL) by others and us and have proven to be valuable tools to facilitate preclinical *in vivo* studies [28–31].

The aim of the present work was to develop an improved preclinical mouse model of AML, broadening and increasing the use and quality of studies performed in the model, by: (i) performing serial retransplantation of primary AML cells to repetitively provide PDX cells for *in vitro* and *in vivo* studies; (ii) introducing genetic engineering of PDX cells to express transgenes using lentiviral transduction; (iii) introducing repetitive and sensitive disease monitoring *in vivo* by bioluminescence imaging (BLI) and; (iv) establishing a stringent set of quality controls to monitor the effect of retransplantation and transgene expression on molecular, phenotypic and functional sample characteristics. Due to these advances, our model system will facilitate future studies on AML biology and novel treatment approaches *in vivo*.

## Methods

### Primary AML cells

Fresh BM or PB samples from adult AML patients were obtained from the Department of Internal Medicine III, Ludwig-Maximilians-Universität, Munich, Germany, during the years 2012 and 2013. Specimens were collected for diagnostic purposes before start of treatment. Written informed consent was obtained from all patients or legal guardians in the cases where patients were minors. The study was performed in accordance with the ethical standards of the responsible committee on human experimentation (written approval by Ethikkommission des Klinikums der Ludwig-Maximilians-Universität, Munich, number 068-08) and with the Helsinki Declaration of 1975, as revised in 2000. Pediatric AML samples were described previously [19].

### Mice

NOD.Cg-*Prkdc* ^*scid*^ *IL2rg* ^*tm1Wjl*^/SzJ (NSG; The Jackson Laboratory, Bar Harbour, ME, USA) were maintained under specific pathogen-free conditions in the research animal facility of the Helmholtz Zentrum München, Munich, Germany. Animals had free access to food and water, and were housed with a 12-hour light–dark cycle and constant temperature. All animal trials were performed in accordance with the current ethical standards of the official committee on animal experimentation (written approval by Regierung von Oberbayern, number 55.2-1-54-2532-95-10). When clinical signs of illness became apparent (more than 60% leukemic cells within PB, rough fur, hunchback, or reduced motility), mice were sacrificed equally in all passages. If leukemia became not apparent, mice were killed and analyzed 25 weeks after cell injection by latest.

### Recovering PDX cells from mice and serial transplantation

From first generation mice harboring primary AML cells (see S1 Methods), PDX cells were reisolated out of femurs, tibiae and spleen by mincing the tissues and filtration through a cell strainer, followed by Ficoll gradient centrifugation in case of splenic cells. PDX AML cells were identified by staining for human CD45, CD33, CD3 and CD19 and flow cytometry analysis (see S1 Methods). Without further enrichment or manipulation, 1x10^6^–1x10^7^ total BM cells were reinjected into next recipient NSG mice for reexpansion (secondary transplantation).

### Targeted next generation deep sequencing

Genetic characterization of patient specimens and PDX cells was performed using a targeted, multiplexed amplicon resequencing approach (Haloplex, Agilent, Boeblingen, Germany). Mutational hotspots or the entire coding sequences, as appropriate, of 43 genes known to be recurrently mutated in myeloid malignancies (S1 Table) were amplified from 250 ng of genomic DNA. The resulting libraries were sequenced on an Illumina MiSeq instrument (Illumina, San Diego, USA) using 2x250 bp paired-end reads. Sequence data were aligned to the human reference genome (version hg19) using BWA [32]. Single nucleotide variants and short insertions or deletions were called using the VarScan 2 [33] and Pindel algorithms [34], respectively, using a custom data analysis pipeline. Known and putative leukemia-associated mutations were identified through a review of published data and public databases including COSMIC and dbSNP, and novel variants were assessed based on their functional consequences on the amino acid level (i.e., nonsense and frameshift mutations were considered pathogenic). A variant allele frequency (VAF) of 2% was used as the threshold for mutation calling.

### Statistical analysis

Mean and standard deviation of the mean were calculated using the Microsoft Excel 2010 software (Microsoft, Redmont, WA, USA). LIC frequencies were calculated according to Poisson statistics using the ELDA software application (http://bioinf.wehi.edu.au/software/elda).

### Additional Methods

Please refer to S1 Methods for description on first engraftment of primary AML cells in NSG mice; monitoring engraftment of PDX cells by flow cytometry analysis of PB; surface antigen characterization of PDX AML cells by flow cytometry; cloning; lentiviral transduction; enrichment of transgene-expressing cells; *in vivo* bioluminescence imaging (BLI); quantification of BLI pictures; and Limiting dilution transplantation assay (LDTA).

## Results

The aim of the present work was to develop the individualized mouse model of AML further and increase its capabilities for future *in vivo* studies on AML biology or treatment efficiency of novel therapeutic strategies.

### High consistency of AML-specific mutations between primary and PDX AML cells

**Fig 1:**
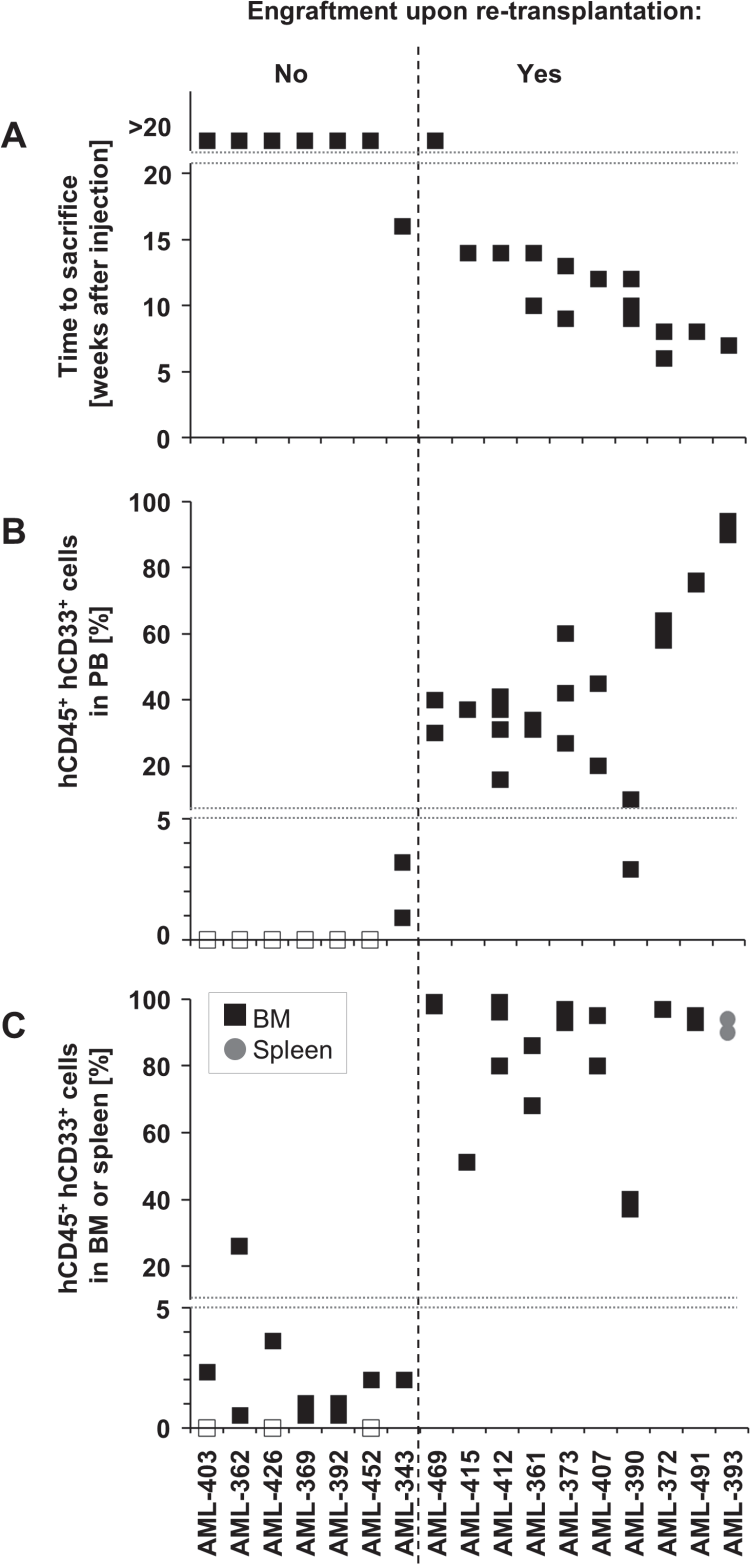
Engraftment of primary AML cells in NSG mice predicts reengraftment capacity. 10^7^ fresh primary AML cells were injected and successfully engrafted in NSG mice; shown are characteristics of the first engraftment regarding passaging time (time period from cell injection until clinical signs of leukemia or latest between 20 and 25 weeks) **(A)**; percentage of cells positive for both hCD45 and hCD33 at time of sacrifice within mouse PB **(B)** and within BM (black cubes) or spleen (grey circles) **(C)**. Each mark visualizes data obtained from a single mouse. Open cubes indicate 0% human cells. Dotted line discriminates samples that reengrafted in secondary recipients from samples that did not. Please refer to S1A Fig. for exemplary FACS plots.

According to published protocols [6,16,17,19,35], we transplanted cells from 29 adult patients with AML. Patients' clinical characteristics are depicted in Table 1. In line with published data, 17/29 (59%) samples engrafted in NSG mice, defined by more than 0.1% hCD45+ hCD33+ cells within BM within 20 weeks after transplantation [17,35–38], although with broad differences regarding *in vivo* growth characteristics (Fig. 1; Table 1; see S1 Results for details).

**Table 1:** Patients’ clinical characteristics.

Sample	Tx	Re-	FAB	Disease	Cytogenetics	NPM1	FLT3	ELN	Sex	Age	PB/	Blasts
		Tx		stage						[y]	BM	[%]
**AML-361**	AML	5*	M4	Initial	Normal	Mut	ITD	Int I	F	40	PB	42
**AML-372**	AML	6*	M0	Relapse	Complex,	WT	WT	Adv	M	42	PB	67
				after SCT	Including −17							
**AML-373**	AML	1	M2	Initial	Normal	WT	WT	Int I	F	79	PB	NA
**AML-390**	AML	3*	M4	Initial	ND	WT	WT	NA	F	62	BM	70
**AML-393**	AML	5*	M4	Relapse	46,XX,	WT	WT	Adv	F	47	BM	54
				after SCT	ins(10;11)							
					(p12;q23q23)							
**AML-407**	AML	1*	M0	Relapse	47,XX,t(4;8)	WT	WT	Adv	F	58	PB	34
				after SCT	(p15;q22),+12							
**AML-412**	AML	4*	M1	Initial	Normal	Mut	ITD	Int I	F	65	BM	96
**AML-415**	AML	2*	NA	Relapse	Normal	Mut	ITD	Int I	F	68	BM	NA
**AML-469**	AML	1*	M4	Initial	47,XY,+8	WT	ITD	Adv	M	25	PB	83
**AML-491**	AML	3*	NA	Relapse	Aberrant	WT	WT	Adv	F	53	PB	44
**AML-343**	AML	0	NA	Initial	Normal	Mut	ITD	Int I	M	79	PB	NA
**AML-362**	AML	0	M4	Initial	Normal	Mut	WT	Fav	M	71	BM	60
**AML-392**	AML	0	M2	Initial	NA	WT	ITD	NA	F	65	BM	25
**AML-396**	AML	0	M4	Initial	Aberrant	WT	WT	Adv	M	73	BM	42
**AML-403**	AML	0	M1	Initial	46,XX,	WT	WT	Adv	F	33	BM	80
					del(12)(p13)/							
					48,XX,+3,+18							
**AML-426**	AML	0	NA	Relapse	Normal	WT	ITD	Int I	F	55	PB	29
**AML-452**	AML	0	M1	Initial	Normal	Mut	ITD	Int I	F	82	PB	93
**AML-353**	B	NA	M5A	Initial	Complex	Mut	WT	Adv	M	74	PB	46
**AML-405**	B	NA	M1	Initial	NA	Mut	ITD	NA	M	51	PB	89
**AML-410**	T	NA	M4	Initial	Normal	Mut	WT	Fav	M	35	BM	47
**AML-397**	T	NA	NA	Initial	47,XY,+8	WT	WT	Adv	M	73	PB	42
**AML-342**	None	NA	M1	Initial	Normal	Mut	ITD	Int I	F	82	PB	69
**AML-345**	None	NA	M1	Initial	Normal	WT	ITD	Int I	F	54	PB	90
**AML-348**	None	NA	M4	Initial	Normal	Mut	ITD	Int I	M	58	BM	60
**AML-391**	None	NA	NA	Initial	47,XY,+13	WT	WT	Adv	M	62	BM	46
**AML-401**	None	NA	M1	Initial	46,XY,t(11;14)	Mut	WT	Adv	M	73	PB	78
					(q13;q32)							
**AML-421**	None	NA	M1	Initial	Normal	Mut	WT	Fav	F	47	BM	67
**AML-433**	None	NA	NA	Initial	Normal	Mut	WT	Fav	F	48	BM	67
**AML-441**	None	NA	NA	Relapse	Complex	WT	WT	Adv	M	43	BM	78

Tx (nature of human cells isolated after initial transplantation); Re-Tx (amount of passages of successful re-transplantation)

* (next passage in progress during manuscript preparation); T (T cells); B (B cells); FAB (French–American–British classification system); SCT (stem cell transplantation); NPM1 (nucleophosmin-1); WT (wildtype); Mut (mutated); FLT3 (Fms-related tyrosine kinase 3); ITD (internal tandem duplication); ELN (European LeukemiaNet classification system); Fav (favorable); Int I (intermediate I); Adv (adverse); F (female); M (male); PB (peripheral blood); BM (bone marrow); NA (not available); ND (not determined).

**Fig 2:**
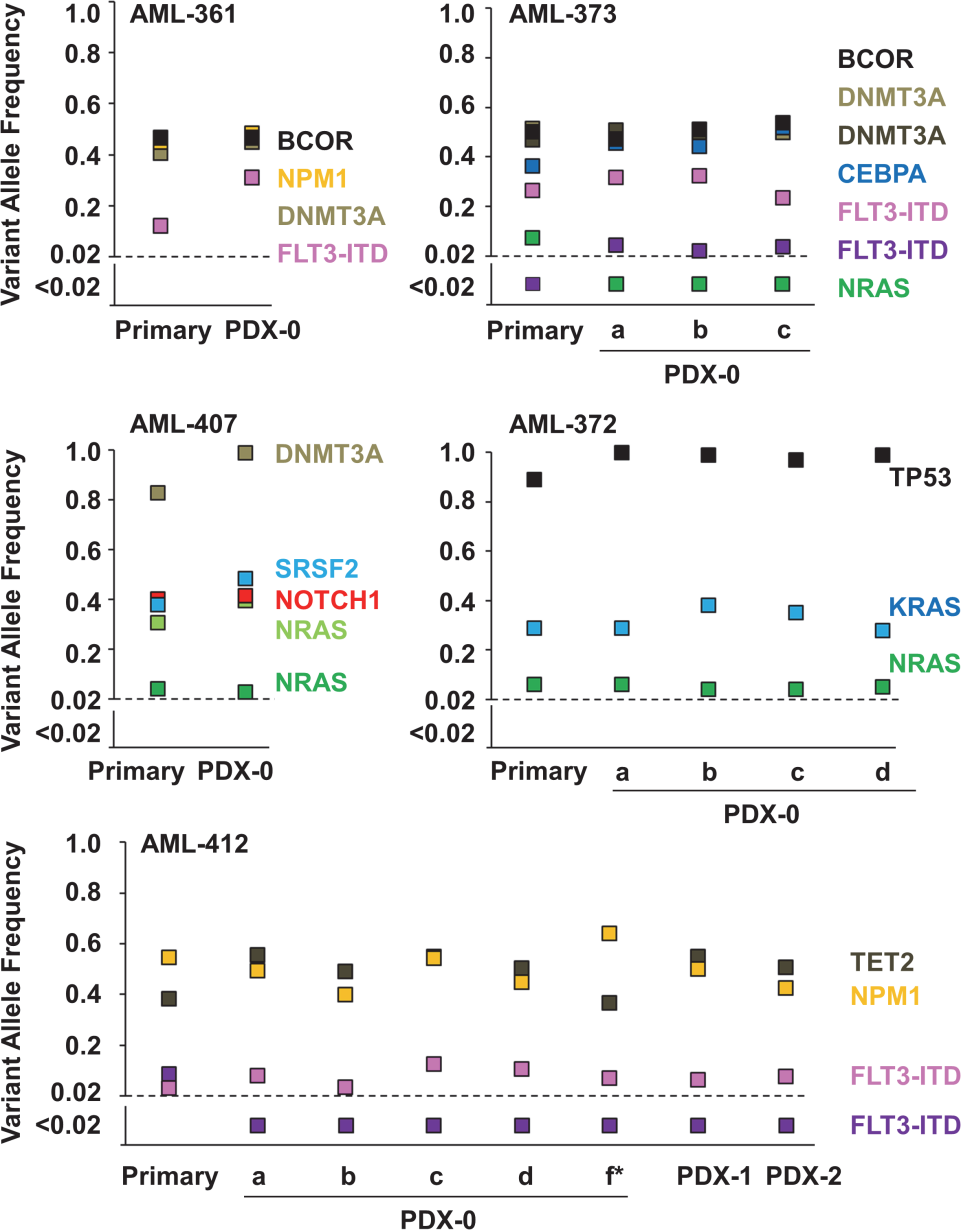
Engraftment and retransplantation of AML cells in NSG mice conserves genetic alterations of the primary sample. Primary AML patient samples and matched PDX cells, reisolated out of the BM (CD45 chimerism 80–99%) after first passage in NSG mice (PDX-0) or after 1 or 2 re-transplantation cycles (PDX-1/-2), were characterized by targeted resequencing of 43 AML-related genes (S1 Table). Plots depict variant allele frequencies for each driver gene mutation found within the sample. a/b/c/d/f: PDX cells of three to five mice injected in parallel were analyzed. *: primary cells were frozen and thawed before injection. *BCOR* (BCL-6 corepressor); *CEBPA* (CCAAT/enhancer binding protein alpha); *DNMT3A* (DNA (cytosine-5)-methyltransferase 3 alpha); *FLT3* (Fms-related tyrosine kinase 3); ITD (internal tandem duplication); *KRAS* (Kirsten rat sarcoma viral oncogene homolog); *NPM1* (nucleophosmin-1); *NRAS* (neuroblastoma RAS viral oncogene homolog); *SRSF2* (serine/arginine-rich splicing factor 2); *TET2* (tet methylcytosine dioxygenase 2); *TP53* (tumor protein p53). Raw data is depicted in S2 Table.

**Fig 3:**
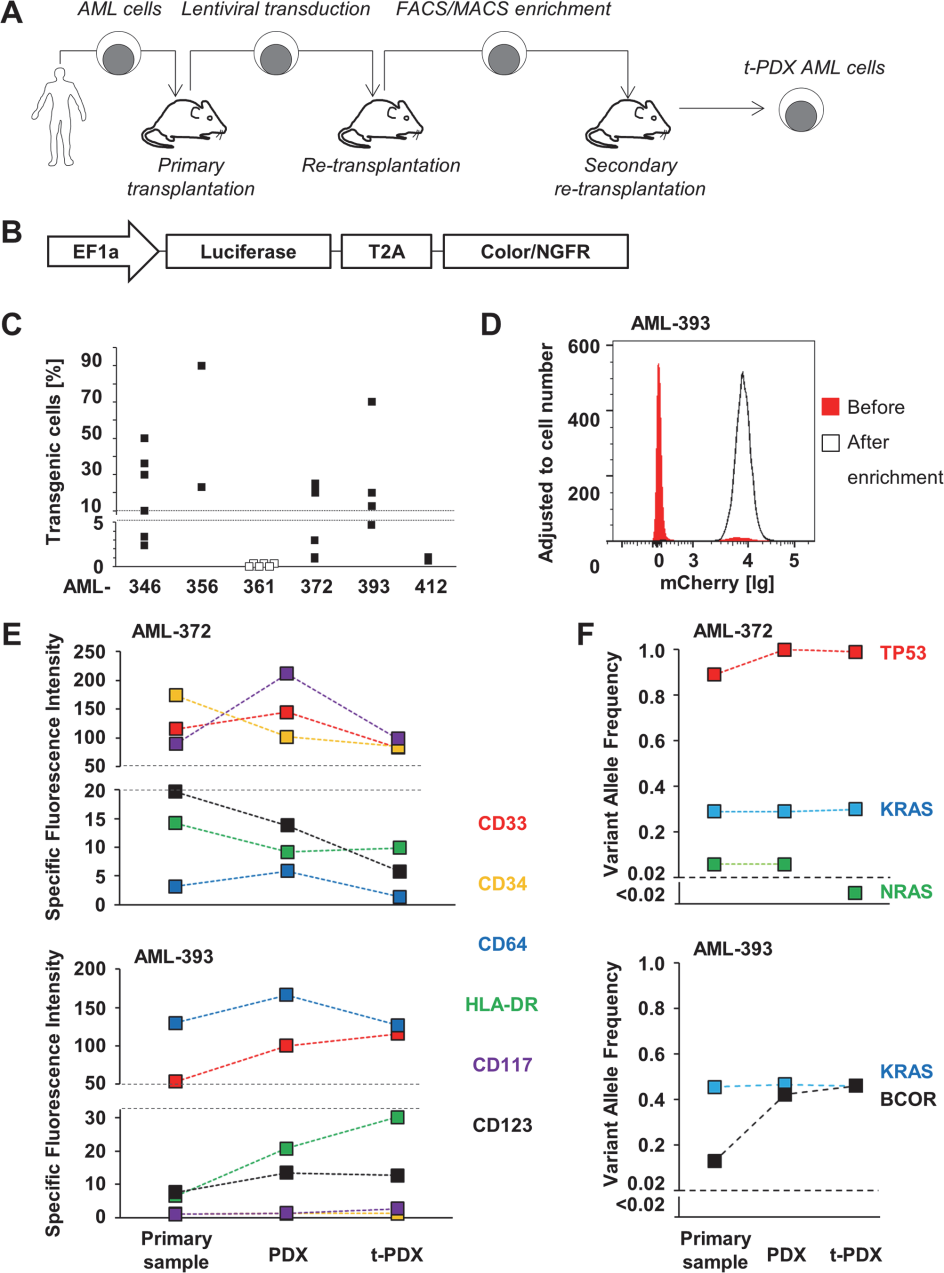
PDX AML cells allow genetic engineering without altering molecular sample characteristics. **(A)** Scheme of the process of generating transgenic PDX (t-PDX) AML cells. PDX cells were transduced after first or second retransplantation cycle. **(B)** Scheme of the vector constructs. **(C)** Transduction rate in t-PDX AML cells after lentiviral transduction and cell amplification in mice was measured by FACS analysis of fluorochrome or NGFR expression. Each mark visualizes data obtained from a single transduction. Open mark: no transgenic cells were detectable. **(D)** Enrichment of transgenic cells using flow cytometry was measured using mCherry expression after cell amplification in mice. **(E)** Genetic engineering does not alter immunophenotype; primary cells, untransduced PDX cells after fourth retransplantation and enriched transgenic t-PDX cells were analyzed by multicolor flow cytometry; specific fluorescence intensity is depicted. See also S3C Fig. for exemplary FACS plots of AML-372. Raw data is depicted in S3 Table. **(F)** Genetic engineering does not markedly alter AML-specific mutations; genomic DNA was isolated out of primary cells, untransduced PDX cells and enriched transgenic t-PDX cells; VAF of mutations was profiled by targeted resequencing. *BCOR* (BCL-6 corepressor); *KRAS* (Kirsten rat sarcoma viral oncogene homolog); *NRAS* (neuroblastoma RAS viral oncogene homolog); *TP53* (tumor protein p53). Raw data is depicted in S2 Table.

AML is a genetically heterogeneous group of diseases, and various gene mutations have been shown to be associated with treatment response and patient outcome [39]. Furthermore, AML patients may harbor multiple, genetically related disease subclones [40,41]. The benefit of using patient-derived cells in preclinical *in vivo* models depends on their ability to faithfully recapitulate the genotypic heterogeneity of the disease. Thus, we performed comprehensive analyses as quality controls, as proposed recently [41]. Using a targeted resequencing approach that covers 43 genes with known roles in AML pathogenesis (S1 Table), we studied six primary patient samples and matched xenografts. Per sample, we identified two to six mutations (Fig. 2, Fig. 3F; S2 Table). Each patient sample contained mutations with a variant allele frequency (VAF) of close to 50%, indicating that these variants were present in most cells, assuming heterozygosity. All of these mutations, which mark the founding clone of the AML cell population, were preserved in the PDX cells recovered from mice after amplification, consistent with data published previously [41]. In each primary sample, we detected additional mutations with a significantly lower VAF, suggesting the presence of subclones within the AML cell population [41,42]. Most of these mutations were also detected within the respective PDX cells. In two cases, we observed an increase in the VAF of mutations in PDX compared to primary cells, indicating that the subclone carrying the mutation had an engraftment or growth advantage (AML-361 and AML-393; Fig. 2 and Fig. 3F). Four samples showed evidence of polyclonal engraftment. In these cases, variants found in the patient at a low VAF were detected in PDX cells with similar, low allelic frequencies, that were highly consistent between multiple mice transplanted in parallel (AML-372, AML-373, AML-407, and AML-412; Fig. 2). In two samples, small subclones (VAFs of the subclone-specific mutations below 10%) were detected in the patient sample but were undetectable in mice (AML-373 and AML-412). These subclones may have had a relative engraftment or proliferation disadvantage in our model. In one sample from a patient with a *FLT3*-ITD (60bp length), we detected a second, additional *FLT3*-ITD (96 bp length) in PDX cells obtained from three mice injected in parallel (VAF 2–4%). This second mutation was not detectable in the primary specimen (AML-373). Since the same, additional *FLT3*-ITD emerged in all three mice that were inoculated with the patient’s cells in parallel, we conclude that a small subclone carrying this variant was present in the patient sample, albeit below our limit of detection. After xenotransplantation, the subclone carrying the 96bp *FLT3*-ITD expanded relative to the 60bp *FLT3*-ITD-mutated clone, and thus had an engraftment or growth advantage in our model. Our data are in line with published results describing the challenge of reliably mimicking rare subclones upon xenotransplantation [41].

These results illustrate several important characteristics of our model: (i) driver mutations present in the founding clone of primary AML specimens are preserved in xenografts; (ii) certain subclones present within the patients’ primary cell population may have an engraftment or growth advantage or disadvantage in mice, as indicated by the increase or decrease, respectively, of the VAF in PDX cells compared to the patient sample; (iii) in the three samples that were xeno-transplanted in multiple mice in parallel, engraftment characteristics of subclones are reproducible; (iv) PDX cells do not gain mutations in the studied AML driver genes, if they were unmutated in the patient's specimen.

### Serial transplantation of PDX AML cells

Next, capacity of initially engrafted cells to reengraft in next generation recipient mice was analyzed. 10/17 (59%) of established PDX samples, corresponding to 10/29 (34%) of all transplanted samples, reengrafted after first retransplantation. As positive predictive markers for successful repassaging, we identified the following growth characteristics of primary cells in the first transplantation: (i) overt leukemia in mice within 20 weeks after cell injection, accompanied by (ii) a substantial percentage of human cells in mouse BM and (iii) more than 5% leukemic cells in the blood at time of sacrifice (Fig. 1, S1B Fig.). A threshold of 0.1 to 1% human cells within BM to define positive engraftment, as proposed by others [10,17,35–38], was not sufficient to reengraft these cells in our hands, and might be explained by the absence of long-term LICs.

Furthermore, serial transplantation of PDX cells was evaluated, to enable repetitive and reproducible experiments. Samples were retransplanted in up to six consecutive generations of mice. Until now, 6 samples could be analyzed for capacity to reengraft serially for more than three passages. 5/6 (83%) samples engrafted serially in all retransplantation experiments performed (S2 Fig.). Only one specimen (AML-373) did not allow retransplantation for more than one cycle. These results are in line with published data where successful secondary and tertiary engraftment was used as prove for the presence of long-term LICs which were not present in all AML samples [9].

Serial reengraftment of PDX AML samples was further characterized. Similar between all samples, murine BM consisted mainly of human leukemic cells at time of overt illness (minimum 80%, maximum 99.8%). In contrast, other characteristics varied widely between samples, including passaging time (minimum 4 weeks, maximum 15 weeks), percentage of leukemic cells in PB (minimum 1%, maximum 99.8%), or amount of leukemic cells in the spleen (minimum 1x10^6^, maximum 7x10^8^) (S2 Fig.). Splenic enlargement was observed in one sample (AML-393), while all other samples showed only minor infiltration of the spleen. This is in contrast to PDX ALL cells, where splenomegaly represents a common feature between all samples and 1x10^8^ human cells can regularly be reisolated out of the spleen [31], but consistent with other studies analyzing AML engraftment in NSG mice [17].

Serial transplantation might affect clonal composition of PDX cells. Targeted resequencing of serially transplanted cells revealed that they still contained the same leukemia-associated mutations as primary patient specimens and PDX cells after initial engraftment (Fig. 2; S2 Table). In addition, analysis of six AML associated antigens revealed that the immunophenotype of PDX cells after four retransplantations was similar to the primary specimens for most antigens tested (Fig. 3E, S3 Fig.; S3 Table). Thus, serial expansion did not severely alter molecular sample characteristics of AML cells, indicating that PDX cells closely resemble the disease they originate from.

### Genetic engineering in AML PDX cells

Until now, preclinical studies in AML were restricted to established AML cell lines [22–24], although they might inherit unphysiologic mutations acquired upon prolonged *in vitro* culture. The use of PDX AML cells will allow repetitive molecular studies on cells highly related to the patients, but these cells have never been used for genetic engineering so far.

Here, we established lentiviral transduction of PDX AML cells, which we and others had successfully used to genetically modify PDX ALL cells [28–31] or primary AML cells [9] (Fig. 3A), although lentiviral transduction holds the risk of additional untargeted genetic alterations [43]. Lentiviral constructs coding for, e.g., luciferase and the red fluorochrome mCherry or the antigene nerve growth factor receptor (NGFR), were cloned under the control of the EF1-alpha promoter; transgenes were connected by a T2A linker allowing equimolar expression (Fig. 3B).

Four PDX samples from adult AML patients as described above (AML-361, AML-372, AML-393, AML-412) were included into further studies together with two pediatric PDX samples (AML-346, AML-356) that had been engrafted and serially transplanted before [19] (S4 Table). After transduction and reamplification in mice, percentage of transgenic PDX (t-PDX) cells was determined by flow cytometry. For one sample (AML-361), no transgenic cells were detectable after six independent transduction trials. Within the other five samples, transduction efficiency ranged from 1.1% to 90% (Fig. 3C). Although defined factors determining lentiviral transduction efficiencies could not be identified, specific characteristics of samples, constructs and viruses might influence the process. t-PDX cells were enriched by flow cytometry (Fig. 3D) or by magnetic-activated cell sorting (MACs), and were reinjected into another generation of recipient mice (Fig. 3A). To generate samples with a high percentage of transgenic cells (>90%), transgenic cells were enriched by two rounds of cell sorting. As some samples were transduced with a rather low transduction efficiency (Fig. 3C), cell enrichment inherits the threat of clonal selection, as we could not guarantee that all subclones of a sample have been transduced equally. Nevertheless, lentiviral transduction and cell enrichment did not markedly alter sample characteristics like growth behavior and immunophenotype in t-PDX compared to PDX AML cells or primary specimens, respectively (Fig. 3E, S4A Fig.; S3 Table). Furthermore, the mutational pattern within the defined set of AML driver mutations studied and within the resolution applied was not altered in the 2 samples analyzed, apart from a loss of a rare subclone carrying an *NRAS* mutation (VAF 5%) in t-PDX of AML-372 (Fig. 3F; S2 Table). Transgene expression level remained stable over numerous consecutive transplantations (S4 Fig.). In contrast, CMV promoters were silenced in our ALL model within a short period of time (own unpublished data).

Taken together, lentiviral transduction was feasible in 5/6 (83%) PDX samples; both lentiviral constructs allowed successful transduction indicating a more general applicability of lentiviral constructs. Cell enrichment allowed generation of t-PDX cells without markedly altering sample characteristics. Successful genetic engineering in PDX AML cells now opens a broad variety of possible molecular studies.

### Highly sensitive repetitive BLI of t-PDX AML cells in single mice

We next wanted to evaluate transgene expression in PDX cells and used the expression of luciferase as both a convenient example system and an attractive target protein. Expression of luciferase enables *in vivo* bioluminescence imaging (BLI) for sensitive and repetitive disease monitoring in single living mice over time [44]. BLI would greatly improve analysis of preclinical treatment trials in the individualized mouse model of AML. Until now, monitoring of disease development and treatment efficiencies was hampered by the lack of sensitive and repeatable detection procedures.

A codon-optimized form of firefly luciferase (effluc) was used which enables intense light emission for sensitive monitoring [45]. BLI is frequently and successfully used in tumor cell lines to monitor growth or treatment response *in vivo* [46–50] or to monitor engraftment of human hematopoietic stem cells in mice [51]. We and others have recently established BLI to monitor PDX ALL cells growing in mice [28–31], while BLI could not be performed in PDX AML cells so far due to the lack of genetic engineering.

**Fig 4:**
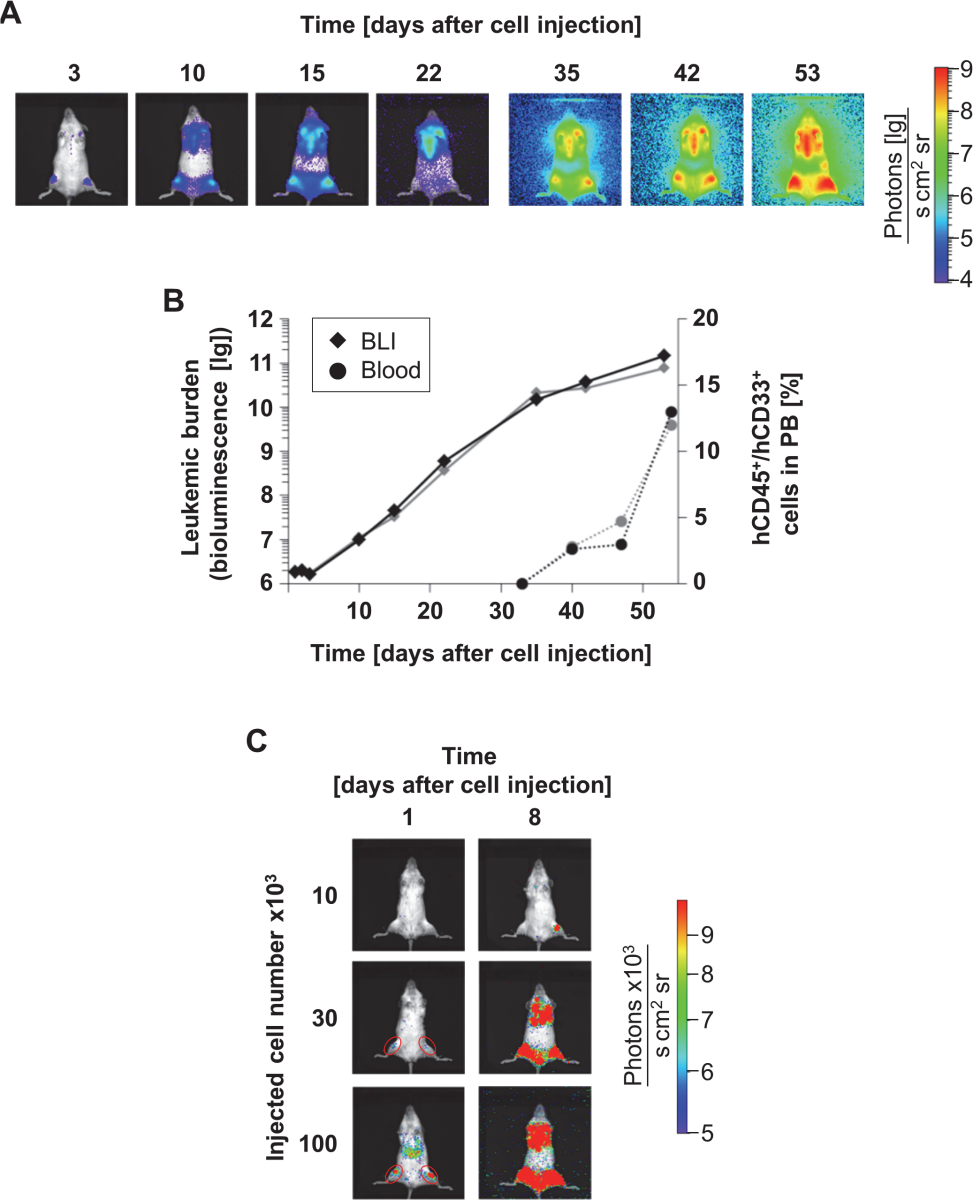
BLI is highly sensitive and reliable in single mice. **(A)** 1x10^5^ t-PDX AML-372 cells were injected into two mice. At indicated days after cell injection, mice were monitored by BLI. Images of one representative mouse are shown. See S5A Fig. for further images. **(B)** BLI signals from the kinetic in **A** were quantified in both animals (diamonds); cells positive for both hCD45 and hCD33 in PB were analyzed in parallel (circles). **(C)** t-PDX AML-372 cells were injected into three mice per group at absolute numbers indicated; 1 and 8 days after cell injection, mice were monitored by BLI; images are shown of one representative mouse per group.

1x10^5^ t-PDX cells of AML-372 were injected into mice and repetitively monitored using BLI (Fig. 4A, S5A Fig.). One day after cell injection, positive BLI signals were visible in the liver and the lower extremities most probably emitted by the BM (S5A Fig.). BLI signal persisted and increased within the legs over time. The liver signal observed shortly after cell injection vanished after another day and might be due to AML blasts that had intermediately homed to the liver, but left the liver again. After ten days, BLI allowed detection of signal in the upper extremities and the sternum. Only at late time points close to animal death, BLI visualized t-PDX AML cells in the spleen (Fig. 4A), reproducing our experience that splenic involvement is difficult to visualize in BLI [31].

When BLI signals were quantified, signal intensity increased exponentially over time implying logarithmic growth of PDX AML cells in mice, which is identical to results obtained for ALL [31]. Over time, BLI signal increased by more than four orders of magnitude, enabling a broad range of BLI signals for sensitively discriminating different AML tumor burdens. By using BLI, we were able to visualize AML growth in mice markedly earlier than by flow cytometry measurement in PB, where human cells were detectable not before six weeks after cell injection (Fig. 4B). Furthermore, BLI using effluc-t-PDX cells seems to be more sensitive and covers a broader range than imaging applying Alexa Fluor 680-labeled monoclonal antibodies [52].

To determine the sensitivity of BLI, different amounts of t-PDX AML cells were injected and mice were imaged as early as 18 hours after cell injection. Application of 1x10^5^ (3/3 mice) and 3x10^4^ (2/3 mice) t-PDX AML cells resulted in a positive BLI signal at both femurs, whereas 1x10^4^ cells yielded no detectable signal (Fig. 4C). Thus, PDX AML cells seem to home to the mouse BM within few hours. Assuming that (i) each mouse has about 3x10^8^ BM cells in total (see supplemental results for calculation of this value) and that (ii) within 18 hours from injection 100% of injected t-PDX cells had successfully homed to the BM, but (iii) cells had not yet started to proliferate, BLI was able to visualize less than 30000 leukemia cells per mouse, hence one human cell among 10000 murine BM cells (0.01%). As homing efficiency is unlikely to reach 100%, BLI probably is even more sensitive.

Thus, expression of luciferase in PDX AML cells enables repetitive BLI for highly sensitive visualization of t-PDX cells in mice at tumor burdens which range within the different definitions of minimal residual disease in AML patients [53], to analyze homing efficiency of PDX cells into the BM niche, and for convenient monitoring of leukemia development *in vivo*.

### BLI for quantifying leukemia stem cell surrogates

AML represents a stem cell disease, and various scientific questions require quantification of leukemia initiating cells (LIC) as stem cell surrogates [20]. The gold standard method for determining LIC frequency is the limiting dilution transplantation assay (LDTA) [6,36,37,54–57]. LDTAs are usually quantified by post mortem analysis of numerous mice on the same day, e.g., 16 weeks after cell injection, and thus evaluating a single time point. We validated the use of BLI for monitoring LDTA over time.

**Fig 5:**
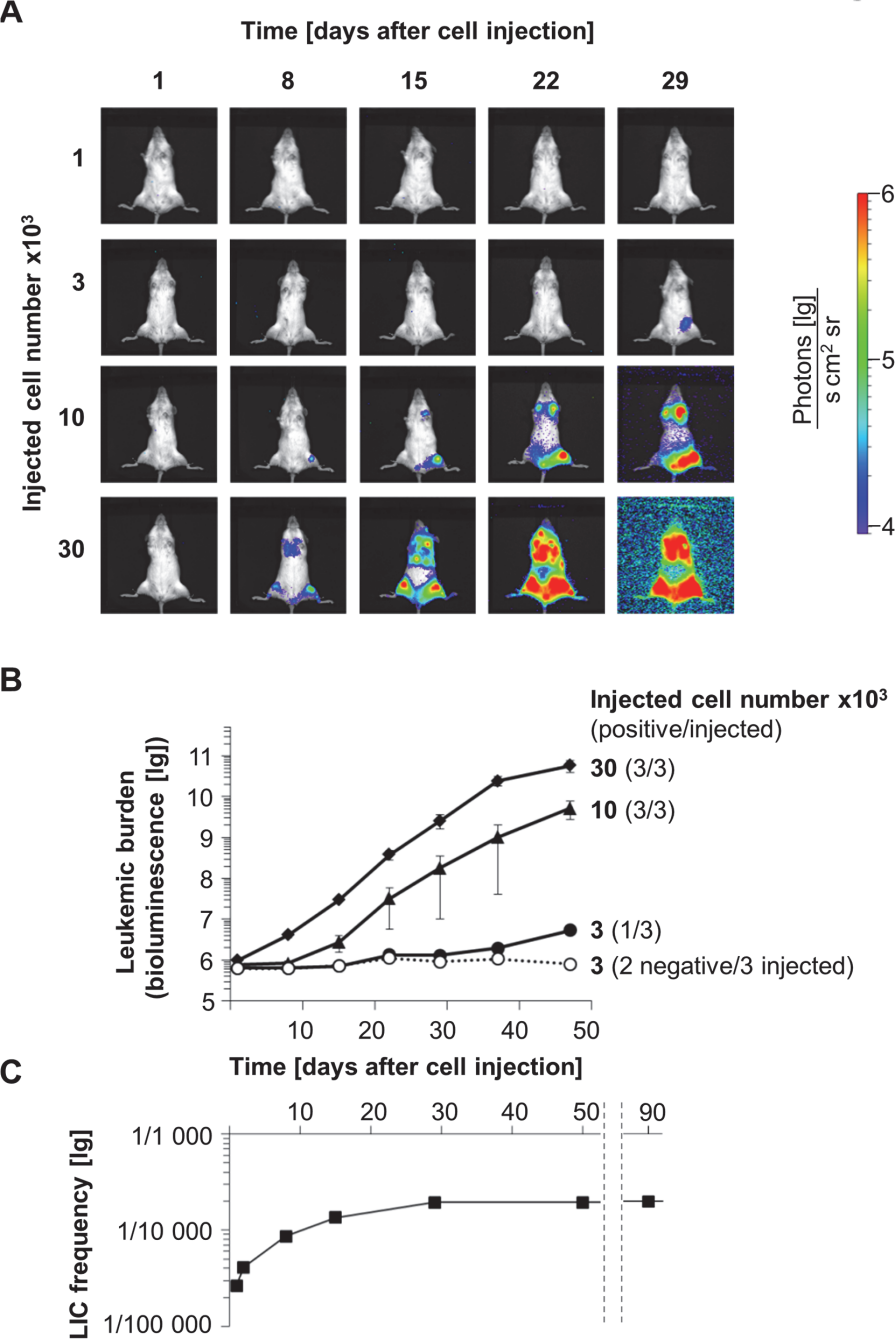
BLI facilitates quantifying leukemia stem cell surrogates. After limiting dilution of t-PDX AML-372 (98% hCD45+ hCD33+ mCherry+), cells were injected into three mice per group (12 mice total) at absolute cell numbers indicated; leukemia development was monitored by BLI over time. **(A)** Images of one representative mouse per group. See S5B Fig. for further images. **(B)** BLI signals from the kinetic in **A** were quantified. Shown are mean values plus/minus standard deviation. **(C)** LIC frequency was calculated at each time point using ELDA software. Raw data is depicted in S5 Table.

Limiting numbers of t-PDX AML-372 cells were injected into groups of mice and repetitive BLI was performed in all animals (Fig. 5A). Reproducing results obtained before, 2/3 mice receiving 3x10^4^ cells were positive in BLI one day after cell injection (S5B Fig.). Seven days later, 2/3 mice of the next dilution (1x10^4^ cells) became positive in BLI; at day 29 after cell injection, all mice that would ever engraft were positive in BLI (Fig. 5A-B; S5 Table). Calculation of LIC frequency showed a clear time-dependent increase, which remained stable as early as four weeks after cell injection (Fig. 5C). Similar results were obtained in t-PDX AML-346 cells (S6 Fig.; S6 Table). LIC frequencies were 1 in 5.1x10^3^ (AML-372) and 1 in 2.8x10^3^ (AML-346), consistent with data obtained from sorted primary AML samples [56]. Thus, BLI enabled reliable determination of LIC frequencies in LDTAs within short periods of time.

## Discussion

In the present work, we developed the individualized xenograft mouse model of AML further by evaluating serial transplantation and introducing lentiviral transduction and bioluminescence imaging. As these technical advances did not markedly alter important sample characteristics such as AML-specific mutations and growth behavior, the advanced model now represents a highly attractive tool for future *in vivo* trials on AML biology and novel treatment strategies.

The individualized xenograft mouse model of AML has been used in the past with a focus on primary transplantation and analysis of stem cell surrogates [25,36,38,57–60]. Retransplantation experiments have mainly been performed to verify self-renewal capacity of engrafted cells [9,10,16,17,19,37,56], or to analyze stability of gene expression profiles [19], but not for studies on AML biology or therapy. In parallel to the successful use of individual ALL PDX cells in preclinical trials [28–31,54], our data suggest broadening the use of the individualized AML mouse model for work on serially transplanted PDX cells. We suggest, however, to quality control PDX cells in comparison to the primary specimen and between different passages concerning AML-characteristic mutations, immunophenotype and functional characteristics like passaging time and organ-specific engraftment rates, as some alterations in mutational or antigen expression patterns might occur during (serial) engraftment.

The value of using the individualized mouse model as preclinical model depends on the capability of PDX cells to faithfully mimic the heterogeneity of the disease. Published results showed that PDX cells resemble the primary samples concerning gene expression profiles [10,11,15,19]. Accordingly, in our hands, AML sample-specific characteristics like surface antigen expression or growth behavior in mice remained mainly stable upon serial transplantation and lentiviral transduction. Recently, genetic stability of xenografted AML cells has been analyzed in detail [41]. The authors saw that founding clone mutations were preserved in PDX cells, but that subclonal architecture was often not reflecting the primary sample. The authors proposed that xenotransplantation models should be controlled by characterizing the genotype of AML cells both before and after xenotransplantation. Therefore, we performed targeted resequencing, to reveal if genetic alterations in PDX AML cells are comparable to the primary sample, not only upon first engraftment as studied recently [41], but also after serial transplantation and after genetic engineering. In agreement with published data [41], we found that AML driver gene mutations present in the founding clone of the AML cell population were preserved in PDX cells, but skewing of the subclonal architecture occurred in certain samples. Of note, we observed polyclonal engraftment in several patient samples, and the size of engrafting subclones (estimated from the VAF of subclonal mutations) was highly reproducible when multiple mice were injected in parallel. Furthermore, certain subclones appear to have engraftment advantages or disadvantages and might be lost or expand upon engraftment, either due to evolutionary procedures also present in patients or due to altered selection pressure factors within the mouse compared to the human microenvironment. From our data, we conclude that current mutations that characterize the founding clone are faithfully recapitulated upon xenotransplantation in mice and can be reliably studied in preclinical trials.

Serially transplantable PDX cells offer important advantages compared to existing traditional AML cell lines, as cell lines may not be representative for the heterogeneity of AML. PDX cells might resemble the wide variety of genetic subgroups within AML and thus serve as clinically relevant model for drug testing. Furthermore, serial transplantation of PDX cells allows repetitive and reproducible analyses with stable and defined patient samples, both for *in vitro* and *in vivo* applications. However, three of the six samples allowing serial transplantation were from patients with adverse-risk genetic features who had suffered relapse after allogeneic transplantation, while the other three were from patients with newly diagnosed, ELN “Intermediate-I” risk group AML. All but one patient died within twelve months after sample collection. Therefore and as a limitation of the model, aggressive AMLs derived from adverse-risk patients might be especially suitable for allowing serial transplantation in NSG mice, consistent with data concerning initial engraftment capacity [11,19].

So far, molecular studies in AML were restricted to AML cell lines, as PDX AML cells were not systematically generated and genetic engineering was never performed in PDX AML cells. Our protocol for lentiviral transduction of PDX AML cells will allow multiple molecular studies, to gain a deeper and clinically more relevant understanding of AML biology.

So far, using the individualized xenograft mouse model of AML for preclinical treatment trials was hampered by the inability to sensitively and repetitively monitor disease progression. Drug effects were determined post mortem [27], or at single time points using BM aspirates [25,26]. We introduced BLI as convenient and quantitative *in vivo* readout parameter for PDX AML cells. BLI enabled sensitive, reliable and repetitive disease monitoring and reduces required mouse numbers. BLI represents a convenient, simple and fast method to quantify stem cell frequencies.

In conclusion, we advanced the xenograft mouse model of patient-derived AML by introducing and controlling serial transplantation, genetic engineering and *in vivo* imaging. As characterized patient cells are available repetitively and can be monitored *in vivo* in a highly sensitive way, the improved model will enable detailed reproducible *in vivo* studies on AML biology and therapy in the future.

## Supporting Information

S1 Fig Reengraftment capacity is predicted by degree of initial engraftment. (A) Exemplary FACS plots for staining of hCD33 hCD45 are presented for sample AML-372 PB (d61), BM and spleen (both d66). (B) 17 of 29 samples engrafted in NSG mice, defined by more than 0.5% hCD45+ hCD33+ cells within BM within 20 weeks after transplantation. 10^7^ cells were re-injected into next generation recipients and percentage of hCD45+ hCD33+ cells within BM within 20 weeks was measured. Each mark visualizes data obtained from one patient sample.
(PDF)

S2 Fig Serial transplantation of PDX AML cells. (A) PDX AML cells which successfully engrafted in first recipients were re-isolated and serially transplanted into further recipient mice. Waved closure: in progress at time of manuscript preparation; black line: no re-engraftment could be observed after first re-transplantation in AML-373. (B) Serial transplantation of PDX samples was analyzed regarding passaging time (defined as time period from cell injection until animal death due to leukemia) and percentage or absolute number of cells positive for both hCD45 and hCD33 at time of sacrifice within mouse PB, BM, or spleen, respectively. Each mark visualizes data obtained from a single mouse. Cross: Not determined.
(PDF)

S3 Fig Molecular stability of PDX AML cells. (A) Immunophenotype of primary cells and PDX cells after four retransplantation cycles was analyzed by multicolour flow cytometry; specific fluorescence intensity of six AML associated antigens and of the aberrantly expressed antigen CD7 (AML-361) is depicted. Raw data is depicted in S3 Table. (B) Difference in SFI of six antigens analyzed of PDX cells after four retransplantation cycles or transgenic PDX cells and primary specimens is depicted. ND: not determined. (C) Exemplary FACS plots for staining of AML related antigens are presented for sample AML-372; for each antigen, isotype control (grey lines) and specific staining (colored lines) is shown.
(PDF)

S4 Fig Characterization of t-PDX AML cells. (A) t-PDX AML cells show similar growth behavior as non-transgenic PDX cells. t-PDX cells (white bars) were compared to PDX cells (black bars) regarding passaging time and percentage or absolute number of cells positive for both hCD45 and hCD33 at time of sacrifice within mouse PB or spleen, respectively; shown are means +/- standard deviation. (B) Transgene expression remains stable over passaging. Expression of mCherry was analyzed by flow cytometry directly after cell enrichment by flow cytometry and cell amplification in mice, and after additional one, two or four retransplantation cycles.
(PDF)

S5 Fig Highly sensitive repetitive BLI of t-PDX AML-372 cells in single mice. Additional time points and reduced color scale to visualize lower BLI signals for the growth kinetics shown in Fig. 4A (A) and Fig. 5A, 3x10^4^ cells (B).
(PDF)

S6 Fig BLI for quantifying leukemia stem cell surrogates. LDTA was performed in AML-346 and monitored by BLI as depicted in S6 Table. If BLI showed a positive signal in two independent measurements, mice were sacrificed as engraftment was proven. BLI-negative animals were followed up until day 98, when mice were sacrificed; no hCD45 and hCD33 expressing cells were detected by flow cytometry in BM of BLI negative mice. LIC frequency was quantified at each time point using ELDA software.
(PDF)

S1 Methods (PDF)

S1 Results (PDF)

S1 Table Genes analyzed by targeted next generation deep sequencing. (PDF)

S2 Table Variant Allele Frequencies of mutations in AML specimens and PDX cells. (PDF)

S3 Table Immunophenotype of primary specimens and matched PDX cells. (PDF)

S4 Table Clinical characteristics of pediatric AML patients. (PDF)

S5 Table Ratio of BLI-positive mice after indicated time points after AML-372 injection. (PDF)

S6 Table Ratio of BLI-positive mice after indicated time points after AML-346 injection. (PDF)
