# Supplementary material for: An Advanced Preclinical Mouse Model for Acute Myeloid Leukemia Using Patients' Cells of Various Genetic Subgroups and In Vivo Bioluminescence Imaging
Source: PLoS One. 2015 Mar 20;10(3):e0120925. doi: 10.1371/journal.pone.0120925 (PMC4368518; doi:10.1371/journal.pone.0120925)
Supplement: S1 Fig — (A) Exemplary FACS plots for staining of hCD33 hCD45 are presented for sample AML-372 PB (d61), BM and spleen (both d66). (B) 17 of 29 samples engrafted in NSG mice, defined by more than 0.5% hCD45+ hCD33+ cells within BM within 20 weeks after transplantation. 107 cells were re-injected into next generation recipients and percentage of hCD45+ hCD33+ cells within BM within 20 weeks was measured. Each mark visualizes data obtained from one patient sample. (PDF) [file pone.0120925.s001.pdf]

S1

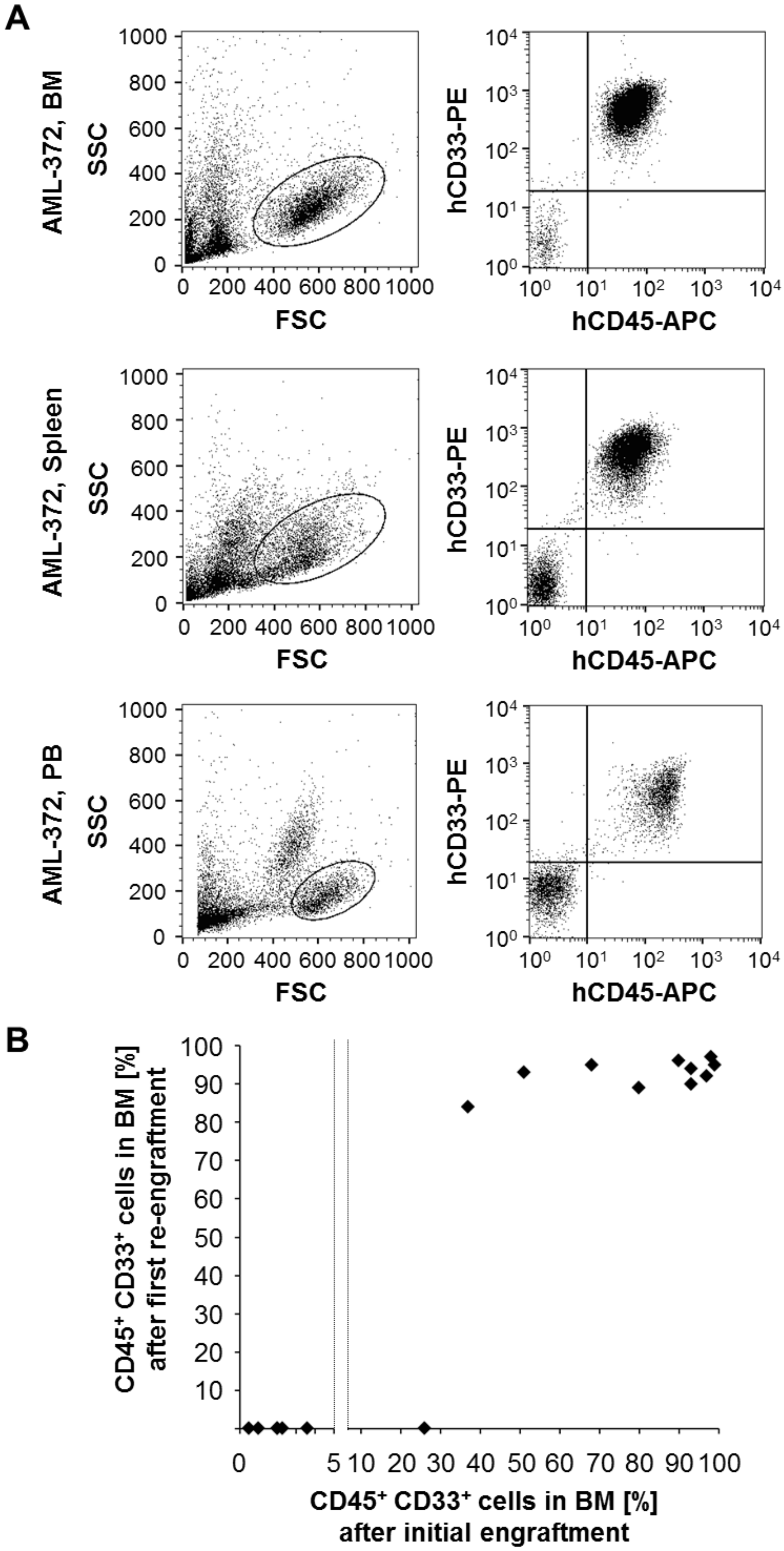
A
AML-372, BM
AML-372, Spleen
AML-372, PB
SSC
FSC
hCD33-PE
hCD45-APC
B
CD45⁺ CD33⁺ cells in BM [%]
after first re-engraftment
CD45⁺ CD33⁺ cells in BM [%]
after initial engraftment

Figure S1: Reengraftment capacity is predicted by degree of initial engraftment. (A) Exemplary FACS plots for staining of hCD33 hCD45 are presented for sample AML-372 PB (d61), BM and spleen (both d66). (B) 17 of 29 samples engrafted in NSG mice, defined by more than 0.5% hCD45+ hCD33+ cells within BM within 20 weeks after transplantation. 10^7 cells were re-injected into next generation recipients and percentage of hCD45+ hCD33+ cells within BM within 20 weeks was measured. Each mark visualizes data obtained from one patient sample.
