# Supplementary material for: An Advanced Preclinical Mouse Model for Acute Myeloid Leukemia Using Patients' Cells of Various Genetic Subgroups and In Vivo Bioluminescence Imaging
Source: PLoS One. 2015 Mar 20;10(3):e0120925. doi: 10.1371/journal.pone.0120925 (PMC4368518; doi:10.1371/journal.pone.0120925)
Supplement: S2 Fig — (A) PDX AML cells which successfully engrafted in first recipients were re-isolated and serially transplanted into further recipient mice. Waved closure: in progress at time of manuscript preparation; black line: no re-engraftment could be observed after first re-transplantation in AML-373. (B) Serial transplantation of PDX samples was analyzed regarding passaging time (defined as time period from cell injection until animal death due to leukemia) and percentage or absolute number of cells positive for both hCD45 and hCD33 at time of sacrifice within mouse PB, BM, or spleen, respectively. Each mark visualizes data obtained from a single mouse. Cross: Not determined. (PDF) [file pone.0120925.s002.pdf]

S2

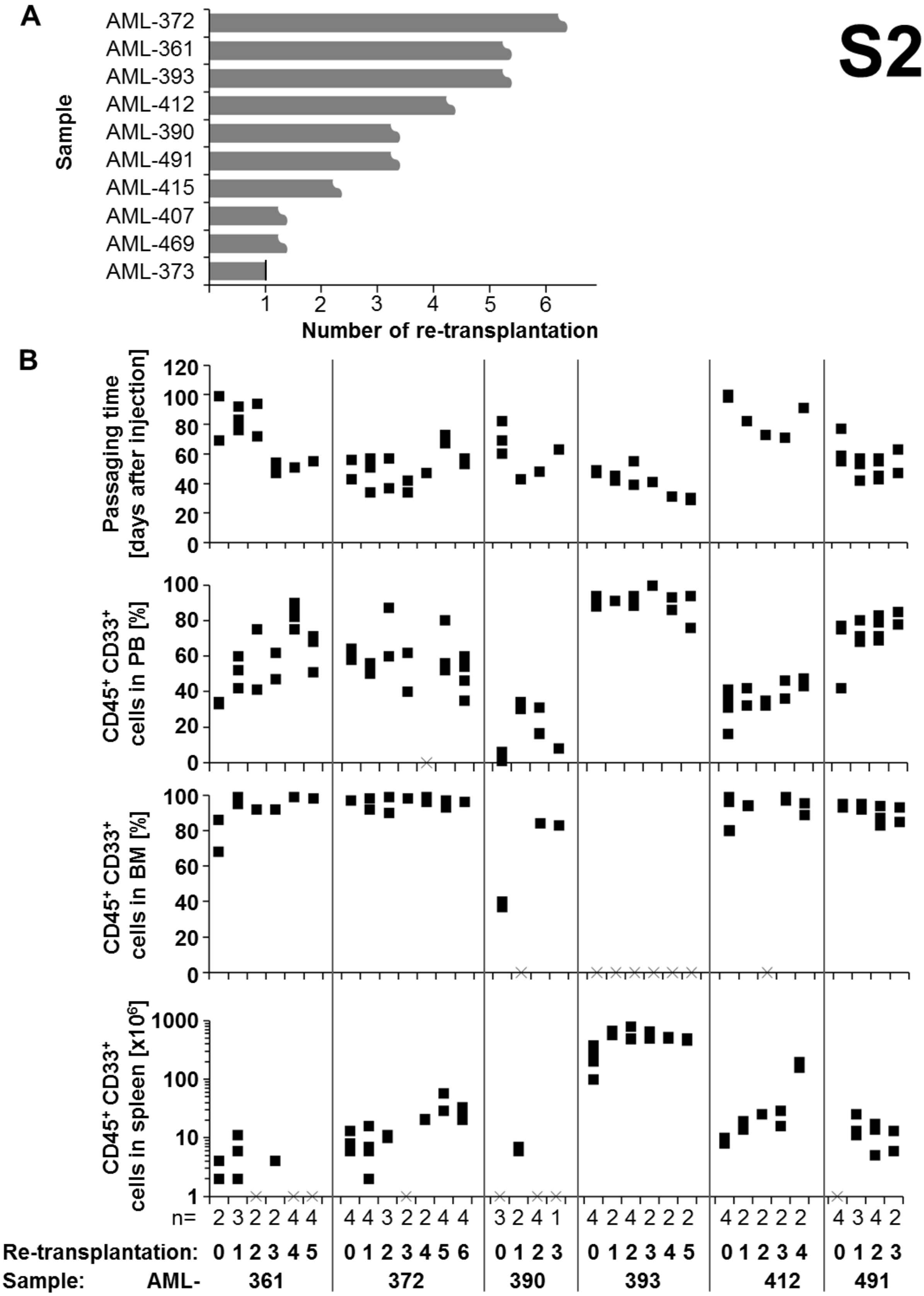
A
AML-372
AML-361
AML-393
AML-412
AML-390
AML-491
AML-415
AML-407
AML-469
AML-373
Sample
1 2 3 4 5 6
Number of re-transplantation
B
Passaging time [days after injection]
0 20 40 60 80 100 120
CD45+ CD33+ cells in PB [%]
0 20 40 60 80 100
CD45+ CD33+ cells in BM [%]
0 20 40 60 80 100
CD45+ CD33+ cells in spleen [x10^6]
1 10 100 1000
n= 2 3 2 2 4 4 4 4 3 2 2 4 4 3 2 4 1 4 2 4 2 2 2 4 2 2 2 2 4 3 4 2
Re-transplantation: 0 1 2 3 4 5 0 1 2 3 4 5 6 0 1 2 3 0 1 2 3 4 5 0 1 2 3 4 0 1 2 3
Sample: AML- 361 372 390 393 412 491

Figure S2. Serial transplantation of PDX AML cells. (A) PDX AML cells which successfully engrafted in first recipients were re-isolated and serially transplanted into further recipient mice. Waved closure: in progress at time of manuscript preparation; black line: no re-engraftment could be observed after first re-transplantation in AML-373. (B) Serial transplantation of PDX samples was analyzed regarding passaging time (defined as time period from cell injection until animal death due to leukemia) and percentage or absolute number of cells positive for both hCD45 and hCD33 at time of sacrifice within mouse PB, BM, or spleen, respectively. Each mark visualizes data obtained from a single mouse. Cross: Not determined.
