# Supplementary material for: An Advanced Preclinical Mouse Model for Acute Myeloid Leukemia Using Patients' Cells of Various Genetic Subgroups and In Vivo Bioluminescence Imaging
Source: PLoS One. 2015 Mar 20;10(3):e0120925. doi: 10.1371/journal.pone.0120925 (PMC4368518; doi:10.1371/journal.pone.0120925)
Supplement: S3 Fig — (A) Immunophenotype of primary cells and PDX cells after four retransplantation cycles was analyzed by multicolour flow cytometry; specific fluorescence intensity of six AML associated antigens and of the aberrantly expressed antigen CD7 (AML-361) is depicted. Raw data is depicted in S3 Table. (B) Difference in SFI of six antigens analyzed of PDX cells after four retransplantation cycles or transgenic PDX cells and primary specimens is depicted. ND: not determined. (C) Exemplary FACS plots for staining of AML related antigens are presented for sample AML-372; for each antigen, isotype control (grey lines) and specific staining (colored lines) is shown. (PDF) [file pone.0120925.s003.pdf]

S3

A

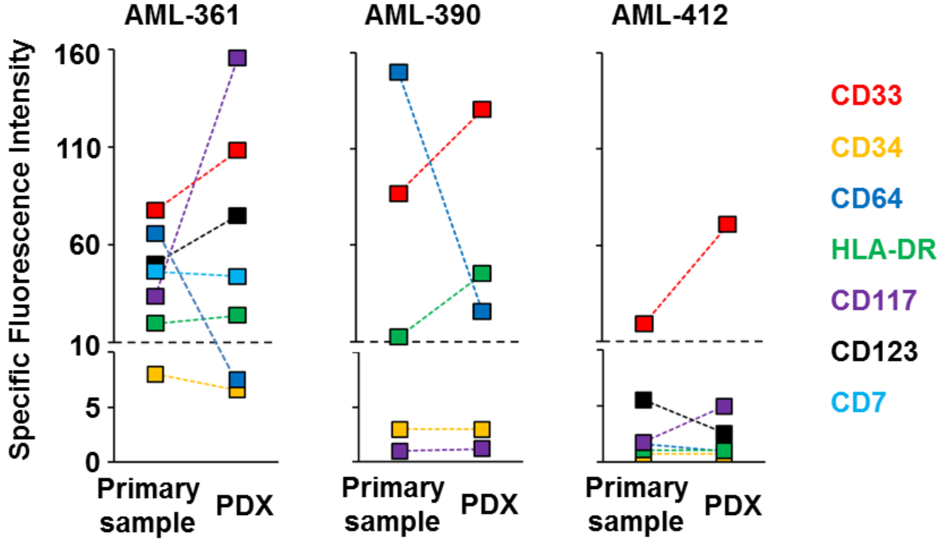

B

AML-	361	372	t-372	390	393	t-393	412
CD33	31	29	-32	44	47	62	52
CD34	-1	-72	-89	0	0	0	0
CD64	-58	3	-2	-125	36	-3	-1
HLA-DR	4	-5	-4	33	14	24	0
CD117	122	122	9	0	0	2	3
CD123	25	-6	-14	ND	6	5	-3

-125	-100	-50	0	50	100	125

Difference in SFI of PDX or t-PDX cells compared to primary specimen

C

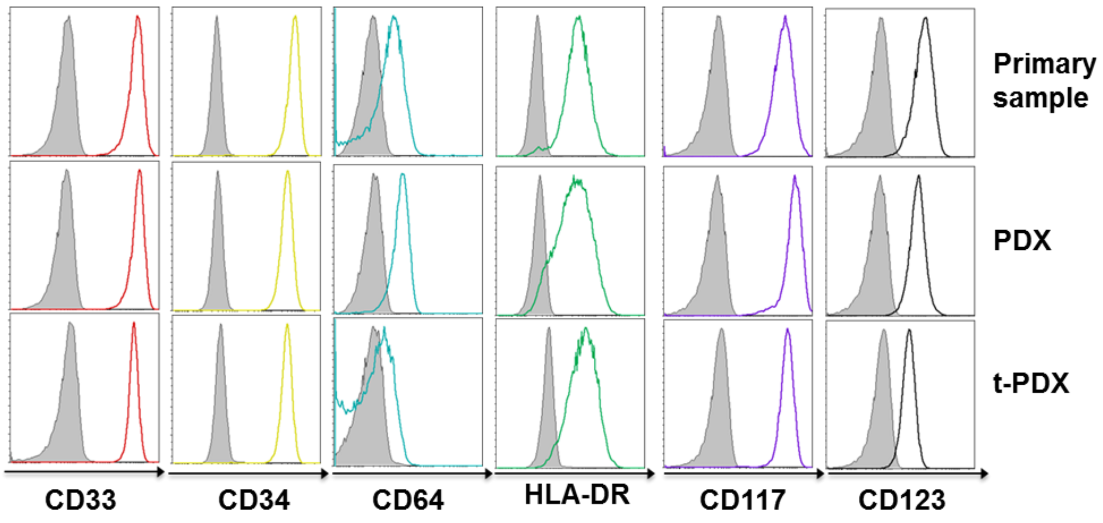

Figure S3. Molecular stability of PDX AML cells. (A) Immunophenotype of primary cells and PDX cells after four retransplantation cycles was analyzed by multicolour flow cytometry; specific fluorescence intensity of six AML associated antigens and of the aberrantly expressed antigen CD7 (AML-361) is depicted. Raw data is depicted in Table S3. (B) Difference in SFI of six antigens analyzed of PDX cells after four retransplantation cycles or transgenic PDX cells and primary specimens is depicted. ND: not determined. (C) Exemplary FACS plots for staining of AML related antigens are presented for sample AML-372; for each antigen, isotype control (grey lines) and specific staining (colored lines) is shown.
