# Supplementary material for: An Advanced Preclinical Mouse Model for Acute Myeloid Leukemia Using Patients' Cells of Various Genetic Subgroups and In Vivo Bioluminescence Imaging
Source: PLoS One. 2015 Mar 20;10(3):e0120925. doi: 10.1371/journal.pone.0120925 (PMC4368518; doi:10.1371/journal.pone.0120925)
Supplement: S4 Fig — (A) t-PDX AML cells show similar growth behavior as non-transgenic PDX cells. t-PDX cells (white bars) were compared to PDX cells (black bars) regarding passaging time and percentage or absolute number of cells positive for both hCD45 and hCD33 at time of sacrifice within mouse PB or spleen, respectively; shown are means +/- standard deviation. (B) Transgene expression remains stable over passaging. Expression of mCherry was analyzed by flow cytometry directly after cell enrichment by flow cytometry and cell amplification in mice, and after additional one, two or four retransplantation cycles. (PDF) [file pone.0120925.s004.pdf]

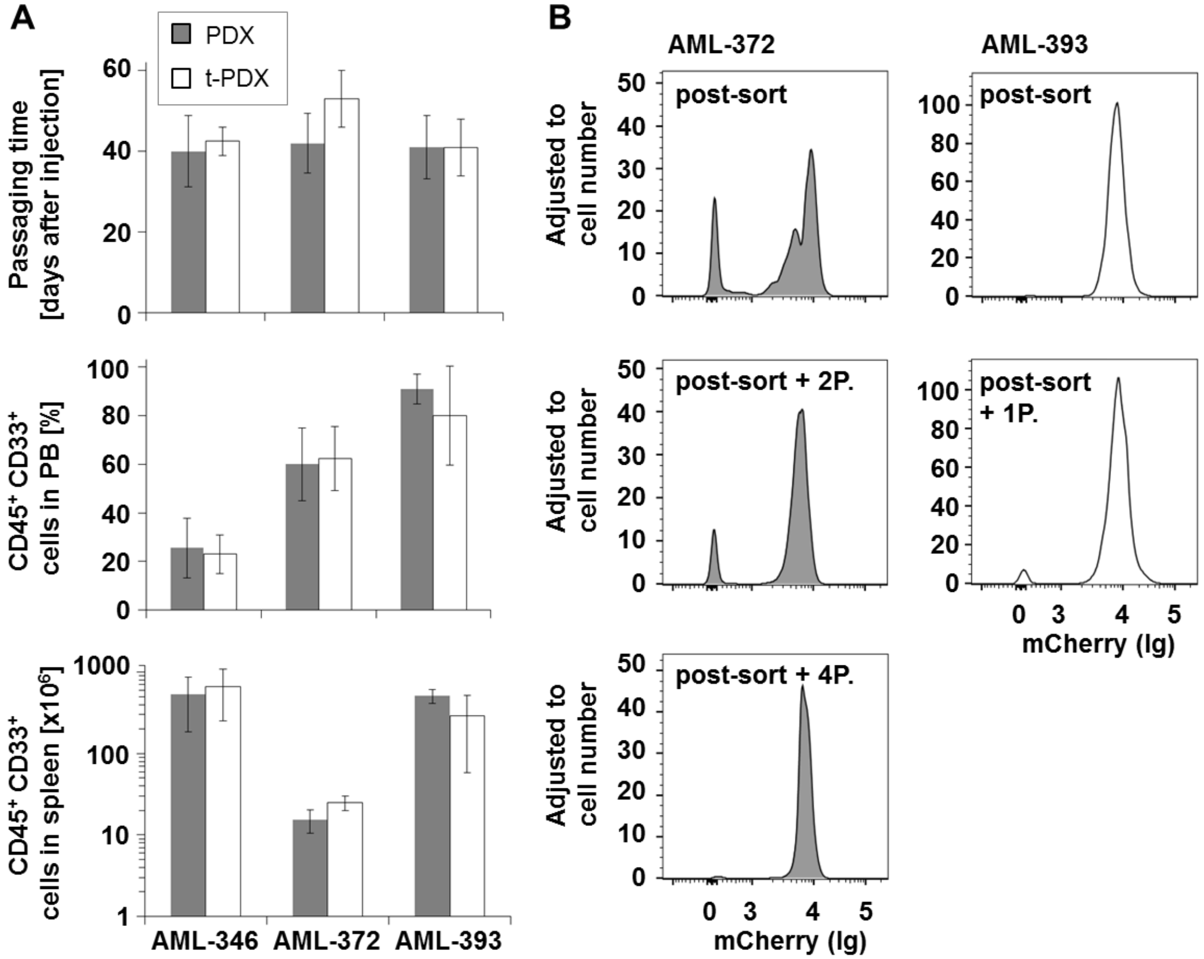

Figure S4. Characterization of t-PDX AML cells. (A) t-PDX AML cells show similar growth behavior as non-transgenic PDX cells. t-PDX cells (white bars) were compared to PDX cells (black bars) regarding passaging time and percentage or absolute number of cells positive for both hCD45 and hCD33 at time of sacrifice within mouse PB or spleen, respectively; shown are means +/- standard deviation. (B) Transgene expression remains stable over passaging. Expression of mCherry was analyzed by flow cytometry directly after cell enrichment by flow cytometry and cell amplification in mice, and after additional one, two or four retransplantation cycles.
