# Supplementary material for: An Advanced Preclinical Mouse Model for Acute Myeloid Leukemia Using Patients' Cells of Various Genetic Subgroups and In Vivo Bioluminescence Imaging
Source: PLoS One. 2015 Mar 20;10(3):e0120925. doi: 10.1371/journal.pone.0120925 (PMC4368518; doi:10.1371/journal.pone.0120925)
Supplement: S5 Fig — Additional time points and reduced color scale to visualize lower BLI signals for the growth kinetics shown in Fig. 4A (A) and Fig. 5A, 3x10^4^ cells (B). (PDF) [file pone.0120925.s005.pdf]

S5

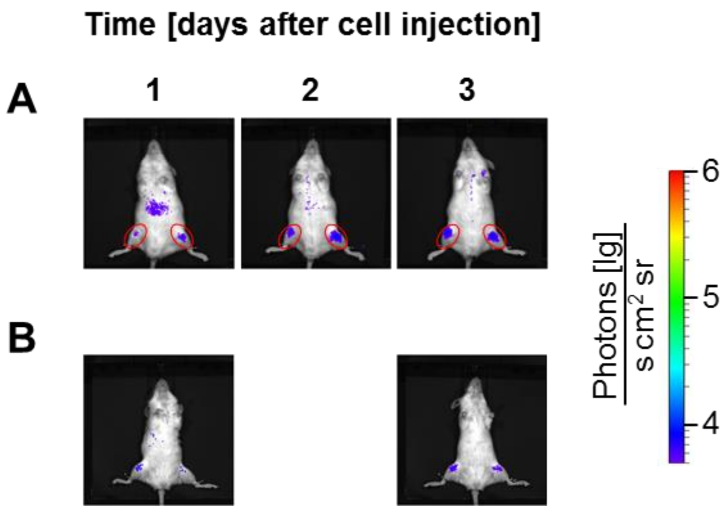

Figure S5. Highly sensitive repetitive BLI of t-PDX AML-372 cells in single mice. Additional time points and reduced color scale to visualize lower BLI signals for the growth kinetics shown in printed Figure 4A (A) and printed figure 5A, $3x10^4$ cells (B).
