# Supplementary material for: An Advanced Preclinical Mouse Model for Acute Myeloid Leukemia Using Patients' Cells of Various Genetic Subgroups and In Vivo Bioluminescence Imaging
Source: PLoS One. 2015 Mar 20;10(3):e0120925. doi: 10.1371/journal.pone.0120925 (PMC4368518; doi:10.1371/journal.pone.0120925)
Supplement: S6 Fig — LDTA was performed in AML-346 and monitored by BLI as depicted in S6 Table. If BLI showed a positive signal in two independent measurements, mice were sacrificed as engraftment was proven. BLI-negative animals were followed up until day 98, when mice were sacrificed; no hCD45 and hCD33 expressing cells were detected by flow cytometry in BM of BLI negative mice. LIC frequency was quantified at each time point using ELDA software. (PDF) [file pone.0120925.s006.pdf]

AML-346

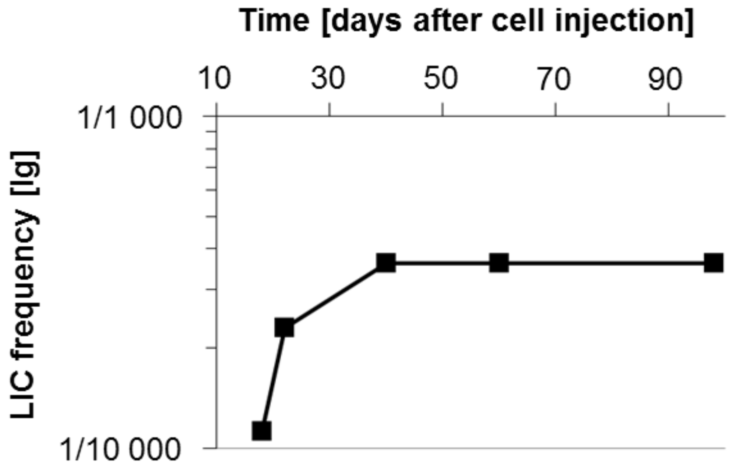

Figure S6. BLI for quantifying leukemia stem cell surrogates. LDTA was performed in AML-346 and monitored by BLI as depicted in Table S6. If BLI showed a positive signal in two independent measurements, mice were sacrificed as engraftment was proven. BLI-negative animals were followed up until day 98, when mice were sacrificed; no hCD45 and hCD33 expressing cells were detected by flow cytometry in BM of BLI negative mice. LIC frequency was quantified at each time point using ELDA software.
