# Supplementary material for: An Advanced Preclinical Mouse Model for Acute Myeloid Leukemia Using Patients' Cells of Various Genetic Subgroups and In Vivo Bioluminescence Imaging
Source: PLoS One. 2015 Mar 20;10(3):e0120925. doi: 10.1371/journal.pone.0120925 (PMC4368518; doi:10.1371/journal.pone.0120925)
Supplement: S1 Methods — (PDF) [file pone.0120925.s007.pdf]

Supplemental Methods

First engraftment of primary AML cells in NSG mice

Human mononuclear cells were obtained out of heparinized BM aspirates or PB by density gradient centrifugation (Ficoll-Paque™ PLUS, GE Healthcare, Uppsala, Sweden). Cells were washed and resuspended in phosphate buffered saline (PBS; Life Technologies, Darmstadt, Germany). $1x10^7$ fresh primary cells were injected into the tail vein of non-irradiated 6 to 16 week old male or female NSG mice.

Monitoring engraftment of PDX cells by flow cytometry analysis of PB

To monitor engraftment of human cells, blood was collected by tail vein aspiration every other week starting from week 6 after cell injection. PB was analyzed by flow cytometry after staining for human CD45 and human CD33 (see below). CD33 positivity was used to confirm AML engraftment. Mice showing any clinical sign of illness (more than 60% leukemic cells within PB, rough fur, hunchback, or reduced motility) were sacrificed by exposure to CO_2. Time from cell injection to animal death due to leukemia was defined as passaging time. If 20 to 25 weeks after cell injection no clinical sign of illness arose, mice were sacrificed and BM was analyzed by flow cytometry. If more than 0.1% of hCD45+ hCD33+ cells were detectable in BM, the sample was classified as engrafted. In four cases, only B cells (hCD45+ hCD33- hCD19+) or T cells (hCD45+ hCD33- hCD3+) were detectable within BM (Table 1).

Surface antigen characterization of PDX AML cells by flow cytometry

Antibodies were added to 50 µL of mouse PB, BM or spleen cell suspensions, and incubated for 30 minutes. For PB, FACS lysing solution (BD, Heidelberg, Germany) was added and samples were incubated for another 15 minutes at room temperature. Cells were washed twice with PBS. The following antibodies were applied: PE mouse anti-human CD33 IgG1 (WM53), APC mouse anti-human CD45 IgG1 (HI30), PE mouse IgG1 Isotype (MOPC-21), APC mouse IgG1 Isotype (MOPC-21; all BD), FITC mouse anti-human CD3 IgG1 (UCHT1), FITC mouse anti-human CD19 IgG1 (HIB19), FITC mouse IgG1 Isotype (MOPC-21), PE/Cy7 mouse anti-human CD271 (NGFR) IgG1

(ME20.4), PE/Cy7 mouse IgG1 Isotype (MOPC-21; all Biolegend, London, UK). Flow cytometry was performed with a FACSCalibur cytometer using CellQuest Pro software (BD). Expression of mCherry and NGFR was determined with an LSRFortessa Cell Analyzer (BD). Flow cytometry data was analyzed using FlowJo software Version 10 (TreeStar Inc., Ashland, OR, USA). Please refer to Figures S1A and S4B for exemplary FACS plots.

Multicolor FACS stainings of patient samples and matched xenograft cells were performed at the Laboratory for Leukemia Diagnostics, Department of Internal Medicine III, LMU, Munich. The median expression intensity of several surface antigens was analyzed by flow cytometry (Navios, Beckman Coulter, Krefeld, Germany), using the following antibodies: CD33 (D3HL60.251), CD34 (581), CD64 (22), HLA-DR (Immu-357), CD117 (104D2D1), CD45 (J33), CD7 (8H8; all Beckman Coulter), and CD123 (7G3; BD Bioscience). Corresponding isotypes were used as controls. A $CD45_{DIM}/SSC_{LOW}$ gate was used to gate on myeloid progenitor cells. Median fluorescence intensity (MFI) values were determined using FlowJo software. Specific fluorescence intensity (SFI = MFI sample/MFI isotype) was calculated as a measure of expression intensity. Samples with SFIs higher than 1.5 were regarded as positive. Only samples with more than 40% viable cells were evaluated. Please refer to Figure S3C for exemplary FACS plots.

Cloning

A codon-optimized form of Firefly luciferase (effluc) [1] was subcloned into the multicloning site of pCDH-EF1-MCS-T2A-copGFP vector (System Biosciences, Mountain View, CA, USA) using EcoRI and BamHI. The 3′ stop codon was removed during PCR amplification. In the same vector, copGFP was replaced by mCherry or nerve growth factor receptor (NGFR), inserted into NsiI - SalI sites.

Lentiviral transduction

Production of lentiviral particles using effluc constructs and lentiviral transduction were performed mainly as described [2]. PDX AML cells were freshly isolated from mouse spleen or BM and re-suspended in RPMI-Medium (Life Technologies) supplemented

with 20% fetal calf serum (Biochrom AG, Berlin, Germany), 5% L-Glutamin, 1% Gentamycin, 1% Penicillin/Streptomycin, 0.6% mixture of rh insulin/human transferrin/sodium selenite (Life Technologies), 1 mM sodium pyruvate, and 50 µM 1-thioglycerole (Sigma-Aldrich, Hannover, Germany). $1x10^6$ cells in 1 ml medium were transferred to a cell culture plate and were transduced overnight with lentiviral constructs in the presence of 8 µg/ml polybrene (Sigma-Aldrich). After 24 h, cells were washed three times with sterile filtered PBS, re-suspended in PBS, and re-injected into next generation recipient mice.

Enrichment of transgene-expressing cells

At advanced leukemic disease, mice were sacrificed, cells were re-isolated and percentage of transgenic (t-) PDX AML cells was analyzed on an LSRFortessa Cell Analyzer. T-PDX AML cells expressing mCherry were sorted on a FACSAria (BD), NGFR expressing cells were enriched by magnetic cell separation using NGFR MicroBeads according to the manufacturer's protocol (Miltenyi Biotech, Bergisch Gladbach, Germany). Enriched cells were re-injected into mice for amplification. Enrichment was repeated once, if necessary, to achieve >90% transgenic cells.

***In vivo* bioluminescence imaging (BLI)**

BLI was mainly performed as previously described [2]. To visualize a recombinant codon-optimized form of firefly luciferase (effluc) [1], D-Luciferin (BIOMOL GmbH, Hamburg, Germany) was injected at 150 mg/kg into the tail vein of mice. Pictures were taken immediately for 30 sec or up to two minutes using a field of view of 12,5 cm with binning 8, f/stop 1 and open filter setting. To monitor tumor growth, mice were typically imaged once or twice weekly.

Quantification of BLI pictures

Quantification of BLI signal was mainly performed as previously described [2]. The Living Image software 4.4 (Caliper Life Sciences, Mainz, Germany) was used for data acquisition and quantification of light emission using a scale with a minimum of $2x10^3$ photons per second per cm^2 per solid angle of one steradian (sr). Different regions of

interest (ROI) were defined and signals were considered positive, when light emission exceeded background in each ROI. Background was measured in mice harboring effluc negative leukemias. A ROI covering the entire animal was used (background $1x10^6$ photons per second). As an exception to determine early engraftment or minimal disease, a small ROI covering the femurs was used (background $3x10^4$ photons per second), as light emission became visible there first. Overt leukemia was considered above 10^{11} photons per second using the ROI covering the entire animal.

Limiting dilution transplantation assay (LDTA)

LDTAs were performed as previously described [3]. t-PDX AML cells were counted and serially diluted in PBS; cells were injected into groups of mice at desired absolute cell numbers. Ratio of BLI positive animals to all animals was used to calculated LIC frequency over time by applying the ELDA software [4]. In AML-346, mice were sacrificed after two positive BLI signals as engraftment was reliably shown.

References

1. Rabinovich BA, Ye Y, Etto T, Chen JQ, Levitsky HI, et al. Visualizing fewer than 10 mouse T cells with an enhanced firefly luciferase in immunocompetent mouse models of cancer. Proc Natl Acad Sci U S A. 2008;105: 14342-14346.

2. Terziyska N, Castro Alves C, Groiss V, Schneider K, Farkasova K, et al. In vivo imaging enables high resolution preclinical trials on patients' leukemia cells growing in mice. PLoS One. 2012;7: e52798.

3. Castro Alves C, Terziyska N, Grunert M, Gundisch S, Graubner U, et al. Leukemia-initiating cells of patient-derived acute lymphoblastic leukemia xenografts are sensitive toward TRAIL. Blood. 2012;119: 4224-4227.

4. Hu Y, Smyth GK. ELDA: extreme limiting dilution analysis for comparing depleted and enriched populations in stem cell and other assays. J Immunol Methods. 2009;347: 70-78.
