# Supplementary material for: An Advanced Preclinical Mouse Model for Acute Myeloid Leukemia Using Patients' Cells of Various Genetic Subgroups and In Vivo Bioluminescence Imaging
Source: PLoS One. 2015 Mar 20;10(3):e0120925. doi: 10.1371/journal.pone.0120925 (PMC4368518; doi:10.1371/journal.pone.0120925)
Supplement: S1 Results — (PDF) [file pone.0120925.s008.pdf]

Supplemental Results

Engraftment of primary AML cells in NSG mice

Different protocols for engrafting primary human AML cells in immuno-compromised mice have been published [1-5]. After experimenting with different details of some of these protocols and as our lab has a long-standing experience in xenografting primary ALL cells [6,7], in our hands an identical handling of primary ALL and primary AML cells turned out most straightforward.

Several steps of our AML protocol might be noteworthy, as they differ from certain published protocols. Regarding the primary sample,

i) only fresh samples were injected directly after aspiration from patients; however, in a single experiment, AML-412 was injected both freshly and after freeze/thawing [8] and time to advanced leukemia did only differ minimally between both cell preparations (fresh: 98-100 days, frozen: 82-124 days);
ii) only samples that were anti-coagulated with Heparin, but not with EDTA, were engrafted, as EDTA seemed to decrease cell viability;
iii) BM and PB samples with any proportion of leukemic blasts were injected, ranging from 34 to 96% (Table 1) to include all available patient samples;
iv) cells were not pre-sorted prior to injection in order to prevent antibody-associated engraftment reduction as reported for CD38 staining [3].

Regarding animal handling,

v) mice were not irradiated prior to injection, as irradiation did not increase engraftment rate in our ALL experiments, but obviously stressed animals;
vi) cells were intravenously (iv) injected into 6 to 16 week old mice as this is technically less challenging compared to neonatal or intrafemoral (if) injections [1,9]; if injections or combined iv plus if injections did not markedly increase AML engraftment in our hands while stressing animals (data not shown).

All mice receiving the same samples in parallel (2 to 4 mice for primary cell injection) showed a similar outcome of engraftment.

Serial transplantation of PDX AML cells

Sample AML-393 was the unique sample showing splenic enlargement and in this sample, spleen-derived PDX AML cells were re-transplanted. In all other samples without splenomegaly, BM-derived PDX AML cells were re-transplanted. In samples AML-361, AML-372, and AML-412, both spleen- and BM-derived cells were once re-transplanted in identical numbers in parallel, and no difference in engraftment time or rate was observed. In contrast to recently published data [1], re-isolation of PDX AML cells out of the liver yielded in low cell numbers in our hands. Re-isolated cells were re-injected either fresh or frozen with similar growth characteristics.

Seven samples did not give rise to PDX AML cells in PB within 20 weeks of incubation, but engrafted in BM to a low extent (1 to 26%, Figure 1A). These cells did not successfully re-engraft upon re-transplantation.

In vivo bioluminescence imaging (BLI)

As our lab is highly experienced in injecting intravenously (iv), iv injection of D-Luciferin was found more convenient than intraperitoneal (ip) injection mainly due to two reasons: (i) reliability of BLI signals was higher after iv compared to ip injection in our hands (data not shown); (ii) after iv injection, imaging could be started earlier after substrate injection compared to ip injection as waiting for substrate redistribution was not required; this allowed shorter incubation times and time periods of anesthesia and less efforts for both animals and experimenters.

Calculation of bone marrow cellularity of NSG mice

Femurs and tibiae of naïve NSG mice were smashed and BM cells were counted. In median, femurs and tibiae contained $43x10^6$ bone marrow cells ($12-84x10^6$, n=22). Percentage of BM cells from femurs and tibiae in different mouse strains are reported to make up about 14% of total BM cellularity [10]. From our measurements, we calculated that NSG mice have around $3x10^8$ bone marrow cells in total.

References

1. Malaise M, Neumeier M, Botteron C, Dohner K, Reinhardt D, et al. Stable and reproducible engraftment of primary adult and pediatric acute myeloid leukemia in NSG mice. Leukemia. 2011;25: 1635-1639.

2. Sanchez PV, Perry RL, Sarry JE, Perl AE, Murphy K, et al. A robust xenotransplantation model for acute myeloid leukemia. Leukemia. 2009;23: 2109-2117.

3. Taussig DC, Miraki-Moud F, Anjos-Afonso F, Pearce DJ, Allen K, et al. Anti-CD38 antibody-mediated clearance of human repopulating cells masks the heterogeneity of leukemia-initiating cells. Blood. 2008;112: 568-575.

4. von Bonin M, Wermke M, Cosgun KN, Thiede C, Bornhauser M, et al. In vivo expansion of co-transplanted T cells impacts on tumor re-initiating activity of human acute myeloid leukemia in NSG mice. PLoS One. 2013;8: e60680.

5. Woiterski J, Ebinger M, Witte KE, Goecke B, Heininger V, et al. Engraftment of low numbers of pediatric acute lymphoid and myeloid leukemias into NOD/SCID/IL2Rcgammanull mice reflects individual leukemogenecity and highly correlates with clinical outcome. Int J Cancer. 2013;133: 1547-1556.

6. Castro Alves C, Terziyska N, Grunert M, Gundisch S, Graubner U, et al. Leukemia-initiating cells of patient-derived acute lymphoblastic leukemia xenografts are sensitive toward TRAIL. Blood. 2012;119: 4224-4227.

7. Terziyska N, Castro Alves C, Groiss V, Schneider K, Farkasova K, et al. In vivo imaging enables high resolution preclinical trials on patients' leukemia cells growing in mice. PLoS One. 2012;7: e52798.

8. Bonnet D. In vivo evaluation of leukemic stem cells through the xenotransplantation model. Curr Protoc Stem Cell Biol Chapter 3. 2008: Unit 3 2.

9. Ishikawa F, Yoshida S, Saito Y, Hijikata A, Kitamura H, et al. Chemotherapy-resistant human AML stem cells home to and engraft within the bone-marrow endosteal region. Nat Biotechnol. 2007;25: 1315-1321.

10. Colvin GA, Lambert JF, Abedi M, Hsieh CC, Carlson JE, et al. Murine marrow cellularity and the concept of stem cell competition: geographic and quantitative determinants in stem cell biology. Leukemia. 2004;18: 575-583.
