# Supplementary material for: An Advanced Preclinical Mouse Model for Acute Myeloid Leukemia Using Patients' Cells of Various Genetic Subgroups and In Vivo Bioluminescence Imaging
Source: PLoS One. 2015 Mar 20;10(3):e0120925. doi: 10.1371/journal.pone.0120925 (PMC4368518; doi:10.1371/journal.pone.0120925)
Supplement: S1 Table — (PDF) [file pone.0120925.s009.pdf]

Table S1: Genes analyzed by targeted next generation deep sequencing

ASXL1	GATA2	NOTCH1	SMC1A
BCOR	GATA3	NPM1	SMC3
BRAF	IDH1	NRAS	SRSF2
CBL	IDH2	PHF6	STAG2
CEBPA	IL7R	PTEN	TET2
DNMT3A	JAK1	PTPN11	TP53
ETV6	JAK2	RAD21	U2AF1
EZH2	JAK3	RUNX1	U2AF2
FBXW7	KIT	SF1	WT1
FLT3	KRAS	SF3A1	ZRSR2
GATA1	MYD88	SF3B1	
