# Supplementary material for: An Advanced Preclinical Mouse Model for Acute Myeloid Leukemia Using Patients' Cells of Various Genetic Subgroups and In Vivo Bioluminescence Imaging
Source: PLoS One. 2015 Mar 20;10(3):e0120925. doi: 10.1371/journal.pone.0120925 (PMC4368518; doi:10.1371/journal.pone.0120925)
Supplement: S2 Table — (PDF) [file pone.0120925.s010.pdf]

Table S2: Variant Allele Frequencies of mutations in AML specimens and PDX cells

Sample	Mutation	Primary	PDX-0					PDX-1	PDX-2	t-PDX
			a	b	c	d	f			
AML-361	DNMT3A: p.R882H	0.41	0.47							
	NPM1:p.L287fs	0.45	0.48							
	FLT3-ITD[a]	0.12	0.31							
	BCOR:p.D551N	0.47	0.47							
AML-372	NRAS:p.Q61K	0.06	0.06	0.04	0.04	0.05				ND
	KRAS:p.G12V	0.29	0.29	0.38	0.35	0.28				0.30
	TP53:p.R209Q	0.89	1.00	0.99	0.97	0.99				0.99
AML-373	NRAS:p.G12S	0.07	ND	ND	ND					
	DNMT3A:p.R882H	0.51	0.46	0.50	0.50					
	DNMT3A:p.S663P	0.47	0.51	0.46	0.53					
	FLT3-ITD[b]	0.26	0.32	0.32	0.23					
	FLT3-ITD[c]	ND	0.04	0.02	0.04					
	CEBPA:p.P23fs	0.36	0.45	0.44	0.52					
	BCOR:p.R810X	0.50	0.47	0.51	0.54					
AML-393	KRAS:p.G12A	0.46	0.47							0.46
	BCOR:p.1012_1013del	0.13	0.42							0.46
AML-407	NRAS:p.Q61H	0.31	0.40							
	NRAS:p.G12V	0.04	0.03							
	DNMT3A:p.R882C	0.83	0.99							
	NOTCH1:p.S1690L	0.40	0.42							
	SRSF2:p.P95H	0.38	0.48							
AML-412	TET2:p.A1097fs	0.38	0.56	0.49	0.55	0.50	0.37	0.55	0.51	
	NPM1:p.L287fs	0.55	0.49	0.40	0.54	0.45	0.64	0.50	0.42	
	FLT3-ITD[d]	0.09	ND	ND	ND	ND	ND	ND	ND	
	FLT3-ITD[e]	0.03	0.08	0.04	0.13	0.11	0.07	0.07	0.08	

Raw data for Figures 2 and 3F. Genomic DNA was isolated out of primary cells and PDX cells after initial engraftment reisolated out of mice injected in parallel (PDX-0 a-f), after 1 or 2 re-transplantation cycles (PDX-1/-2), or after lentiviral transduction and cell enrichment (t-PDX); VAF of mutations was profiled by targeted resequencing of 43 AML-related genes. ND: not detectable (VAF below threshold of 0.02); empty spaces: not determined.

[a] FLT3:p.K602delinsNFYVDFREYEYDLK
[b] FLT3:p.F612delinsKDFREYEYDLKWEFPRENLEF
[c] FLT3:p.E611delinsSGFDGSSDNEYFYVDFREYEYDLKWEFPRENLE
[d] FLT3:p.F612delinsLGPQGSSDNEYFYVDFREYEYDLKWEFPRENLEF
[e] FLT3:p.E611delinsVGDFREYEYDLKWEFPRENLE
