# Supplementary material for: An Advanced Preclinical Mouse Model for Acute Myeloid Leukemia Using Patients' Cells of Various Genetic Subgroups and In Vivo Bioluminescence Imaging
Source: PLoS One. 2015 Mar 20;10(3):e0120925. doi: 10.1371/journal.pone.0120925 (PMC4368518; doi:10.1371/journal.pone.0120925)
Supplement: S3 Table — (PDF) [file pone.0120925.s011.pdf]

Table S3: Immunophenotype of primary specimens and matched PDX cells

Sample	Antigene	Primary	PDX-4	t-PDX
AML-361	CD33	77.7	108.3	
	CD34	8.0	6.6	
	CD64	65.8	7.5	
	HLA-DR	19.8	24.0	
	CD117	33.8	155.8	
	CD123	50.3	75.3	
	CD7	46.4	43.9	
AML-372	CD33	115.6	145.0	83.2
	CD34	173.7	102.1	85.0
	CD64	3.2	5.8	1.4
	HLA-DR	14.2	9.2	9.9
	CD117	89.7	211.7	98.8
	CD123	19.7	13.8	5.8
AML-390	CD33	86.8	130.6	
	CD34	1.2	1.5	
	CD64	150.0	25.5	
	HLA-DR	12.2	45.3	
	CD117	1.0	1.2	
	CD123	20.2	ND	
AML-393	CD33	53.7	100.2	115.9
	CD34	1.1	1.2	1.2
	CD64	130.0	166.3	126.9
	HLA-DR	6.5	20.8	30.3
	CD117	1.0	1.3	2.6
	CD123	7.6	13.5	12.7
AML-412	CD33	19.1	70.8	
	CD34	1.0	1.0	
	CD64	1.6	1.0	
	HLA-DR	1.1	1.0	
	CD117	1.8	5.0	
	CD123	5.5	2.5	

Raw data for Figures 3E and S2A. Immunophenotype of primary specimens, PDX cells after four retransplantation cycles (PDX-4), and after lentiviral transduction and cell enrichment (t-PDX), was analyzed by multicolour flow cytometry; specific fluorescence intensity of six AML associated antigens, and of the aberrantly expressed antigen CD7 (for AML-361), is depicted. ND: not determined.
