# Supplementary material for: An Advanced Preclinical Mouse Model for Acute Myeloid Leukemia Using Patients' Cells of Various Genetic Subgroups and In Vivo Bioluminescence Imaging
Source: PLoS One. 2015 Mar 20;10(3):e0120925. doi: 10.1371/journal.pone.0120925 (PMC4368518; doi:10.1371/journal.pone.0120925)
Supplement: S4 Table — (PDF) [file pone.0120925.s012.pdf]

Table S4: Clinical characteristics of pediatric AML patients

Sample	FAB	Disease stage	Cytogenetics	Mutations	Sex	Age [y]	Outcome	Passage*
**AML-346**	M7	Relapse	Complex	*FLT3* wt, *NPM1* wt	W	1	Died within 12 months	5
**AML-356**	M5	Relapse	Not done	*FLT3* wt, *NPM1* wt	M	5	Died within 12 months	3

* Samples were engrafted previously[1] and introduced in our lab at the indicated retransplantation cycle.

1. Woiterski J, Ebinger M, Witte KE, Goecke B, Heininger V, et al. (2013) Engraftment of low numbers of pediatric acute lymphoid and myeloid leukemias into NOD/SCID/IL2Rcgammanull mice reflects individual leukemogenecity and highly correlates with clinical outcome. Int J Cancer 133: 1547-1556.
