# Supplementary material for: An Advanced Preclinical Mouse Model for Acute Myeloid Leukemia Using Patients' Cells of Various Genetic Subgroups and In Vivo Bioluminescence Imaging
Source: PLoS One. 2015 Mar 20;10(3):e0120925. doi: 10.1371/journal.pone.0120925 (PMC4368518; doi:10.1371/journal.pone.0120925)
Supplement: S5 Table — (PDF) [file pone.0120925.s013.pdf]

Table S5: Ratio of BLI-positive mice after indicated time points after AML-372 injection

Injected cell number $x10^3$	Time [days after cell injection]						
	**1**	**3**	**8**	**15**	**29**	**47**	**89**
**1**	n.a.	n.a.	n.a.	n.a.	0/3	0/3	0/3
**3**	n.a.	0/3	0/3	0/3	1/3	1/3	1/3
**10**	0/3	0/3	2/3	3/3	3/3	3/3	3/3
**30**	2/3	3/3	3/3	3/3	3/3	3/3	†
	**LIC frequency (1 LIC per *n* cells)**						
**Lower limit**	$9.8x10^4$	$6.3x10^4$	$3.0x10^4$	$1.9x10^4$	$1.4x10^4$	$1.4x10^4$	$1.4x10^4$
**Estimate**	$3.8x10^4$	$2.4x10^4$	$1.2x10^4$	$7.4x10^3$	$5.1x10^3$	$5.1x10^3$	$5.1x10^3$
**Upper limit**	$1.4x10^4$	$9.3x10^3$	$4.5x10^3$	$2.8x10^3$	$1.9x10^3$	$1.9x10^3$	$1.9x10^3$

Raw data for Figure 5. After limiting dilution, t-PDX AML-372 cells were injected into three mice per group (12 mice total) at absolute cell numbers indicated; leukemia development was monitored by BLI over time. LIC frequency was calculated at each time point using ELDA software.
