# Supplementary material for: An Advanced Preclinical Mouse Model for Acute Myeloid Leukemia Using Patients' Cells of Various Genetic Subgroups and In Vivo Bioluminescence Imaging
Source: PLoS One. 2015 Mar 20;10(3):e0120925. doi: 10.1371/journal.pone.0120925 (PMC4368518; doi:10.1371/journal.pone.0120925)
Supplement: S6 Table — (PDF) [file pone.0120925.s014.pdf]

Table S6: Ratio of BLI-positive mice after indicated time points after AML-346 injection

Injected cell number $x10^3$	Time [days after cell injection]			
	**18**	**22**	**40**	**98**
**1**	0/4	0/4	1/4	1/4
**10**	3/4	4/4	†	†
**100**	4/4	4/4	†	†
**1,000**	4/4	4/4	†	†
	**LIC frequency (1 LIC per *n* cells)**			
**Lower limit**	$2.8x10^4$	$1.3x10^4$	$8.9x10^3$	$8.9x10^3$
**Estimate**	$8.9x10^3$	$4.3x10^3$	$2.8x10^3$	$2.8x10^3$
**Upper limit**	$2.8x10^3$	$1.5x10^3$	$8.6x10^2$	$8.6x10^2$

Raw data for Figure S6. LDTA was performed in AML-346 and monitored by BLI. If BLI showed a positive signal in two independent measurements, mice were sacrificed as engraftment was proven. LIC frequency was quantified at each time point using ELDA software.
